# phyB and HY5 are Involved in the Blue Light-Mediated Alleviation of Dormancy of *Arabidopsis* Seeds Possibly via the Modulation of Expression of Genes Related to Light, GA, and ABA

**DOI:** 10.3390/ijms20235882

**Published:** 2019-11-23

**Authors:** Marlena Stawska, Krystyna Oracz

**Affiliations:** Department of Plant Physiology, Institute of Biology, Warsaw University of Life Sciences–SGGW, Nowoursynowska str. 159, 02–776 Warsaw, Poland; stawska.marlena@gmail.com

**Keywords:** abscisic acid, cryptochromes, dicot seeds, dormancy alleviation, germination, gibberellins, light, phytochromes, signal transduction, transcription factors

## Abstract

Light is one of the most important environmental factors regulating seed germination. It is known that light inhibits seed germination of some monocotyledonous species and that it is mostly related to the blue wavelength of the spectrum received by cryptochromes (cry). Research has also found that the red light (R) stimulates germination of dicotyledonous seeds and that this reaction involves mainly phytochromes (phy). Surprisingly, up to date, the role and the mechanism of action of blue light (BL) in seed biology of dicot plants is still very poorly understood and some questions are unexplained, e.g., whether BL plays a role in regulation of dicot seeds dormancy and/or germination? If, so what particular elements of light signaling pathway are involved in modulation of this(ese) process(es)? Also, is the BL action in regulation of dicot seeds dormancy and/or germination maybe due to changes of expression of genes related to metabolism and/or signaling of two phytohormones controlling seed-related events, such as gibberellins (GA) and abscisic acid (ABA)? To answer these intriguing questions, the combination of biological, transcriptomic, and genetic approaches was performed in this particular study. The germination tests show that freshly harvested wild type (WT) *Arabidopsis thaliana* Col-0 seeds are dormant and do not germinate in darkness (at 25 °C), while nondormant (after-ripened) seeds germinate well in these conditions. It is also proven that dormancy of seeds of this species is released in the presence of white and/or BL (λ = 447 nm) when placed at 25 °C. Presented here, novel results emphasize the role of BL in dormancy alleviation of dicot seeds, indicating that this wavelength of light spectrum received by phyB induces this process and that the sensitivity to this stimulus depends on the depth of seed dormancy. In addition, it is demonstrated that various elements of phy-mediated pathway can be used in response to the signal induced by BL in germinating dormant seeds of *Arabidopsis*. The quantitative real time PCR analysis supported by results of germination tests of WT, T-DNA insertion mutants (i.e., *hy5*, *hfr1*, and *laf1*) and overexpression transformants of *Arabidopsis* seeds (i.e., *35S:OE:HY5, 35S:OE:HYH*, *35S:OE:HFR1*, and *35S:OE:LAF1*) revealed that the *HY5* gene coding transcription factor is most probably responsible for the control of expression of genes involved in GA/ABA metabolism and/or signaling pathways during BL-dependent dormancy alleviation of *Arabidopsis* seeds, while biological functions of *HYH* and *HFR1* are associated with regulation of germination. The model of BL action in regulation of dormancy alleviation and germination potential of *Arabidopsis* seeds is proposed.

## 1. Introduction

Environmental factors, such as temperature and light, act as clues for plants during their life cycle [1]. The temperature can inform seeds about the season of the year and may influence dormancy induction and/or alleviation, as well as germination [2]. Due to the fact that small seeds (i.e., of *Arabidopsis thaliana*) contain a limited amount of storage materials, the influence of light is particularly important for them, as it can bring crucial information on how deep they are placed in the ground and, thus, if the emerging seedlings will be able to grow into mature plants [3]. In nature, the temperature but also quantity, quality, and direction of light signal depend on the season of the year, time of a day, presence of clouds, and even level of air pollution. In the context of continuously increasing scale of devastation of our planet, these factors are becoming recognized as a source of serious problems. The air pollution consists of particles differing in size and chemical structure determining its properties, such as light absorption and scattering, which in consequence may affect plant growth and development. The changes of the environmental temperature due to global warming influence the mother plant, which in consequence alter seed dormancy and germination behavior of various species [4]. The sensitivity to the light of *Arabidopsis* (ecotype Col-0) seeds characterized by different depth of dormancy is strongly connected with the temperature requirements allowing proper germination [5]. It was reported that, at harvest, this type of seed germinate quite well in darkness, at a range of temperatures from 10 up to 20 °C, but this process is strictly limited at 25 °C. Interestingly, this inhibitory effect of the temperature is alleviated by the presence of white light [5]. Besides the environmental stimuli (i.e., light and temperature), the internal factors (i.e., phytohormones, such as GA and ABA) play a crucial role in modulation of processes occurring in seeds [6,7]. The ABA is a major negative regulator of seed germination, responsible for the induction and maintenance of dormancy, whereas GA have an antagonistic effect to it, as they promote germination. It was proven that the phytohormone contents, signaling, and interactions play important roles in determination of the physiological state of the seed and in regulation of germination process [6,8,9].

*Arabidopsis* plants possess several light sensors including 1) blue light (BL) photoreceptors, such as two phototropins (phot1 and phot2), ZTL/FKF1/LKP2 proteins (ZEITLUPE; FLAVIN–BINDING, KELCH REPEAT, F–BOX 1; and LOV KELCH PROTEIN 2), LLP BL protein (LOV/LOV domain-containing protein, where LOV is called the light, oxygen, or voltage photosensory domain), three cryptochromes (cry; cry1, cry2); 2) red/far red (R/FR) photoreceptors—five phytochromes (phy), such as phyA, phyB, phyC, phyD, and phyE—and 3) UV-B photoreceptor UVR8 [10,11,12,13,14,15,16]. It is known that photoreceptors must be coupled to a biochemical mechanism so that absorption of light energy generates a chemical signal that modifies cellular metabolism and signaling, leading to changes in growth and/or cell differentiation. An increasing number of evidences shows also that signal transduction pathway of light interacts with other paths of endogenous factors, thus possessing a very complicated mechanism of action [6,15]. The phy modulates GA and ABA metabolism and signaling through one of their interacting proteins, PIL5 (PIF3–LIKE 5), that is one of 15 basic helix-loop-helix (bHLH) subfamily members in *Arabidopsis* (including PIFs, PHYTOCHROME INTERACTING FACTORs). It was demonstrated that the seeds of *pil5 Arabidopsis* mutants germinate even under noninductive light condition, whereas the PIL5 overexpressor requires the higher fluence of light for germination [3].

Light perception by phy and cry primarily suppress many protein–protein interactions including CUL4^COP1-SPAs^ E3 ubiquitin ligase (CULLIN 4, CONSTITUTIVE PHOTOMORPHOGENIC 1, and SUPRESS PHYTOCHROME A-105) with key targets, such as transcription factors (i.e., HY5, ELONGATED HYPOCOTYL 5; HYH, HY5–HOMOLOG; HFR1, LONG HYPOCOTYL IN FAR–RED 1; and LAF1, LONG AFTER FAR–RED LIGHT 1), resulting in changes in expression profiles of defined genes [16]. Interestingly, the phy photoreceptors encoded by five genes in *Arabidopsis* (*PHYA*–*PHYE*) seem to partially overlap their functions [10,17]. The phyB regulates gene expression in response to R light, while phyA regulates under continuous far-red (FR) light. It was also proven that phyA is rapidly destabilized upon R light irradiation by E3 ubiquitin ligases including COP1 [18]. The research studies elucidating the role of phy in germination of dicot seeds highlighted that mainly phyB and phyA regulate this process via modulation of expression of genes involved in metabolism of phytohormones, such as GA (i.e., *GA2ox2* and *GA2ox6* (*GA 2*–*OXIDASE 2* and *6*); *GA3ox1* and *GA3ox2* (*GA 3–OXIDASE 1* and *2*)*;* and *KAO2* (*ENT*–*KAURENOIC ACID OXYDASE 2*)), and ABA (i.e., *ABA1* (*ARABIDOPSIS THALIANA ABA DEFICIENT 1*)*; NCED6* and *NCED9* (*NINE*–*CIS EPOXYCAROTENOID DIOXYGENASE 6* and *9*); and *CYP707A2* (*CYTOCHROME P450, FAMILY 707, SUBFAMILY A, POLYPEPTIDE 2*)) [2,3,19]. Moreover, the involvement of GA and ABA in the phy-mediated *Arabidopsis* germination response was also demonstrated [3,20]. It is also known that not only the phytohormone content but also its perception and signal transduction determine seed germinability [3]. For the GA signaling pathway, the most important elements seem to be DELLA factors (including *RGL*—*REPRESSOR OF GA*–*LIKE* genes) and GID1 (GA INSENSITIVE DWARF 1) receptors. The presence of GA enables GID1 activation and formation of the GID1–DELLA complex, which leads to rapid DELLA degradation [21]. The DELLA proteins may also mediate interaction between GA and ABA pathways because one of its targets, XERICO (RING/U-box superfamily protein) regulates ABA metabolism [22]. In plant cells, among the key elements forming the ABA signaling pathway are PYR/PYL/RCAR receptors of ABA (PYRABACTIN RESISTANCE/PYR1–LIKE/REGULATORY COMPONENT OF ABA RECEPTOR); protein phosphatases type 2C (PP2C) encoded by *HAI1*, *2*, and *3* genes (*HIGHLY ABA*–*INDUCED PP2C 1, 2*, and *3*); SNF1-related protein kinases 2 (SnRK2); and ABI transcription factors coded by *ABA INSENSITIVE* genes [23].

Similar to phy, cry also has some diverse and overlapping functions. Although cry signaling pathway seems to be complicated and involves many different signaling proteins, it relies on two main protein–protein interactions. The first leads through cry1 and its interactions with SPA1 protein [24,25]. The SPA1 protein interacts with COP1 in the dark and positively regulates COP1 activity. In addition, COP1 activity may be regulated by interaction with PIF1, which enhances activity of the COP1/SPA complex in darkness [16]. The COP1–SPA1 complex acts as the substrate adaptor of the CUL4^COP1-SPAs^ E3 ubiquitin ligase responsible for ubiquitination and proteasomal degradation of specific proteins, such as HY5, HYH, HFR1, and LAF1 [26]. In consequence, the influence of cry1 on COP1 enables modulation of specific light-related gene expression [25]. The other method of cry action is through their interactions with transcription factors from the CIB (CRYPTOCHROME INTERACTING BASIC HELIX–LOOP–HELIX) family. There are evidences that cry2 by physical interactions with CIB1 and CIB5 can influence, for example, expression of *FT* (*FLOWERING LOCUS T*) and can therefore affect floral initiation in *Arabidopsis* plants [27]. Despite many researches, the physiological function and mechanism of action of cry3 remains unclear and requires future investigation. However, taking into account its biochemical activity in repair of ss-DNA, it was suggested that cry3 may be involved in protection of organellar genomes in *Arabidopsis* against UV damage [14].

The role of the R light and phy in seed biology of dicot plants as well as the BL and cry in seeds germination of monocot species is quite well understood. The BL plays an important role in dormancy induction of freshly harvested cereal grains while the R/FR spectra regulate germinability of many species of dicot seeds including *Arabidopsis* [3,7,20,28,29]. Surprisingly, the function of the BL and light signaling in dormancy and germination of dicot seeds is still very elusive. Therefore, the aim of this study was to characterize the role of BL and particular components of cry/phy-mediated signaling pathways in the regulation of light-dependent germination of dormant *Arabidopsis* seeds. The following research questions were stated: (1) What is the role of BL in regulation of germination of *Arabidopsis* seeds characterized by different depth of dormancy? (2) Which of the cry/phy photoreceptors and/or which elements of the BL/R/FR light signaling pathways participate in the response of the germinating seeds to the BL stimulus? (3) Does the BL-induced signal use particular elements of the phy-mediated pathway? (4) If so, is the regulation of germinability of *Arabidopsis* dormant and/or nondormant seeds by the BL associated with the modulation of the expression of genes related to metabolism and/or signaling of ABA/GA? The biological assays preformed in this study indicated that freshly harvested *Arabidopsis* (Col-0) wild type (WT) seeds were dormant, as they did not germinate in darkness at 25 °C, while the presence of BL and white light had a stimulating effect on this process. To verify whether genes related to phy/cry signaling pathways as well as genes related to GA/ABA metabolism and/or signal transduction might be involved in BL-dependent regulation of dormant *Arabidopsis* seed germination, changes in their relative expression during germination of dormant seeds exposed to different light conditions were investigated using the qRT-PCR method. Obtained results emphasize the role of BL in the regulation of dormancy alleviation of dicot seeds, indicating that cry- and phy- mediated signaling pathways may regulate this process in a light- and dormancy-dependent manner by modulation of transcript levels of genes related to GA/ABA metabolism and signaling pathways. Performed complex transcriptomic analysis and biological tests of WT, T-DNA insertion mutants (i.e., *hy5*, *hfr1*, and *laf1*), and overexpression transformants of *Arabidopsis* seeds (i.e., *35S:OE:HY5, 35S:OE:HYH, 35S:OE:HFR1*, and *35S:OE:LAF1*) highlighted that, from among all genes analyzed in this study, the *HY5* coding transcription factor is most probably responsible for the regulation of expression of genes involved in GA/ABA metabolism and/or signaling pathways during BL-mediated dormancy alleviation of *Arabidopsis* seeds, while two other transcription factors encoded by *HYH* and *HFR1* genes are positive regulators of seed germination.

## 2. Results

### 2.1. The Effect of Blue Light on Germination of Arabidopsis Seeds Characterized by Different Depths of Dormancy

**Figure 1:**
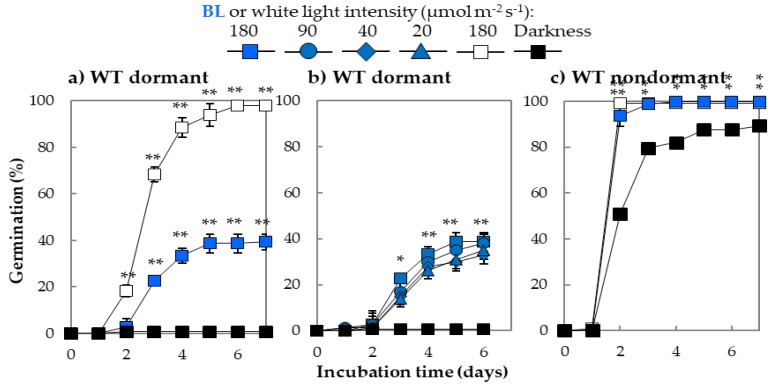
Characteristic of germination of wildtype (WT) *Arabidopsis* (Col-0) seeds: nondormant (**a**) and dormant (**b**,**c**) incubated on water in darkness or under blue (BL) (range of intensities from 20 to 180 µmol m^−2^ s^−1^) or white light (intensity = 180 µmol m^−2^ s^−1^) in a constant temperature of 25 °C: Experiments were conducted in three biological and two technical replicates (100 seeds per each replicate), * and ** indicate the significant differences for *p* ≤ 0.05 and *p* ≤ 0.01 respectively. Results are presented as mean ± SD.

The performed biological test of WT *Arabidopsis* Col-0 seeds characterized by different dormancy levels revealed that freshly harvested seeds were unable to germinate in the dark; thus, they were considered as dormant (Figure 1a,b). The germination of WT dormant seeds was promoted by the constant presence of white light (180 µmol m^−2^ s^−1^), and after 24 h of incubation, more than 90% of seeds were germinated (Figure 1b). Interestingly, the constant exposure of WT dormant seeds to wide range of BL intensities (from 20 up to 180 µmol m^−2^ s^−1^) stimulated germination by around 40% in compare to darkness (Figure 1a,b). Also, dry storage of freshly harvested WT dormant seeds (at room temperature, in darkness) released dormancy, allowing fast germination not only in all applied light conditions—white and BL wavelengths—but also in the dark; hence, they were recognized as nondormant (Figure 1c).

To address the question of whether stimulation of germination of WT dormant seeds requires constant BL illumination and/or shorter treatment, seeds were exposed to BL (180 µmol m^−2^ s^−1^) for different duration: 3, 6, 24, and 48 h (Table S1). The illumination of seeds by BL for 24 and 48 h resulted in similar or even stronger stimulatory effect as that of after continuous exposure to this light condition (Table S1).

Interestingly, 3 and 6 h of exposure of seeds to BL was not sufficient to promote germination of WT dormant seeds and results were similar to these observed in darkness. Moreover, 3 h illumination of WT dormant seeds by BL after 6 h pre–incubation in darkness had strong stimulatory effect on germination while seeds were further incubated without the presence of light—approx. 70% higher germination ratio was observed in compare to constant BL conditions. Surprisingly, the 24 h pre-incubation of seeds in darkness before 3 h illumination by BL had 22% lower stimulatory effect on germination in compare to constant presence of BL (Table S1).

The verification of a possible interaction between BL-induced signal with GA/ABA-related pathways during germination of WT dormant *Arabidopsis* seeds was achieved by the biological tests of WT dormant seeds in the presence of ABA and paclobutrazol (PAC—an inhibitor of GA synthesis) (Table 1). It was observed that the treatment of WT dormant seeds by ABA and PAC solutions significantly decreased the stimulatory effect of BL on dormancy alleviation (Table 1).

### 2.2. qRT-PCR Analysis of Relative Expression of Genes Involved in Light Perception and Signaling During Germination

**Figure 2:**
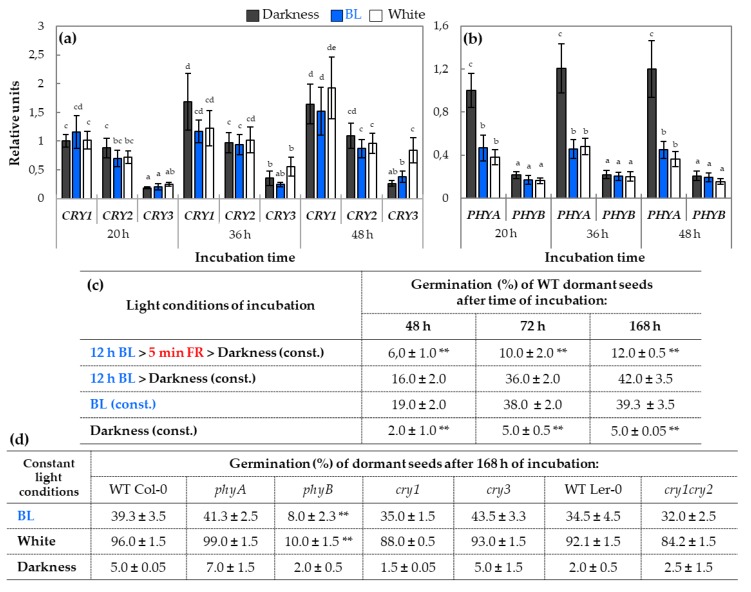
The relative expressions of genes encoding photoreceptors *CRY1*, *CRY2*, and *CRY3* (**a**) and *PHYA* and *PHYB* (**b**) in samples isolated from WT dormant *Arabidopsis* (Col-0) seeds incubated on water for 20, 36, and 48 h in different light conditions (BL and white light of intensity = 180 µmol m^−2^ s^−1^, darkness) at a temperature of 25 °C: The transcript level was normalized to reference genes (*ACT7*, *APC2*, and *HBT*) and to the internal control, which was the expression value of *CRY1* (Figure 2a) or *PHYA* (Figure 2b) obtained in darkness after 20 h of incubation. Three biological and two technical replicates were performed. The bars show the relative units ± SD. The effect of far red (FR) on the regulation of WT dormant *Arabidopsis* (Col-0) seeds germination by BL light (**c**). Seeds were incubated on water in constant temperature of 25 °C at various light conditions. The results are presented as germination % of WT dormant *Arabidopsis* (Col-0) seeds after 48, 72, and 168 h of incubation. For BL-dependent germination assay, seeds were exposed to 5 min of FR light irradiation (intensity = 60 µmol m^−2^ s^−1^) after 12 h of pre-imbibition in the presence of BL (intensity = 180 µmol m^−2^ s^−1^). (**d**) The germination % of WT dormant seeds (in Col-0 background: WT Col–0, *phyA*, *phyB*, *cry1*, and *cry3*; in Ler-0 ecotype: WT Ler-0 and *cry1cry2*) after 168 h of incubation on water in the presence of constant BL and white light (intensity = 180 µmol m^−2^ s^−1^) or in darkness at temperature of 25 °C. Germination assays were performed in three biological and two technical replicates (100 seeds per each replicate). Results are presented as mean ± SD), ** indicates the significant differences for *p* ≤ 0.05 and *p* ≤ 0.01 respectively.

The qRT-PCR analysis preformed on samples isolated from WT dormant *Arabidopsis* seeds incubated under constant BL, white light, or darkness indicated that the expression of genes coding BL receptors cry1 and cry2 was very similar and did not depend on light conditions but equally increased within the duration of germination (Figure 2a). Surprisingly, the expression profile of *CRY3* was different in comparison to two other homologs. In the case of *CRY3*, its expression increased in the presence of white light, while there is no effect observed in darkness either in the presence of BL (Figure 2a). Beside genes that are related mostly to BL perception, the expression of genes encoding R/FR light receptors, *PHYA* and *PHYB*, was also investigated (Figure 2b). The *PHYA* expression was much lower in the presence of BL and white light in compare to darkness, while transcript levels of *PHYB* remained similar in all light conditions during incubation up to 48 h (Figure 2b). To verify if the stimulating effect of BL can be photo-reversed by the FR light, WT dormant seeds were pre-incubated for 12 h in the presence of BL; next, treated with FR light for 5 min; and, then, transferred to darkness. The 5-min FR irradiation of WT dormant seeds previously incubated in the presence of BL for 12 h had a negative effect on germination as 2–3 times less germinated seeds were observed in compare to seeds incubated for 12 h or continuously in the presence of BL (Figure 2c).The possible involvement of cry and phy photoreceptors in the mechanism of BL-mediated dormancy alleviation was attested in germination assay of dormant seeds: WT and mutants of genes encoding particular cry and phy, such as WT Col-0, *phyA*, *phyB*, *cry1*, and *cry3* (in Col-0 background) and WT Ler-0 and *cry1cry2* (Ler–0 ecotype) (Figure 2d).

All tested seeds did not germinate in darkness. The dormancy was alleviated by the constant presence of BL or white light (180 µmol m^−2^ s^−1^) in all tested types of seeds except *phyB*. After 168 h of incubation, approx. 40% and 90% of dormant seeds WT Col-0, *phyA*, *cry1*, and *cry3*, and WT Ler-0 and *cry1cry2* germinated respectively on BL and white light, while in the case of *phyB*, just 8–10% seeds germinated in these conditions (Figure 2d).

**Figure 3:**
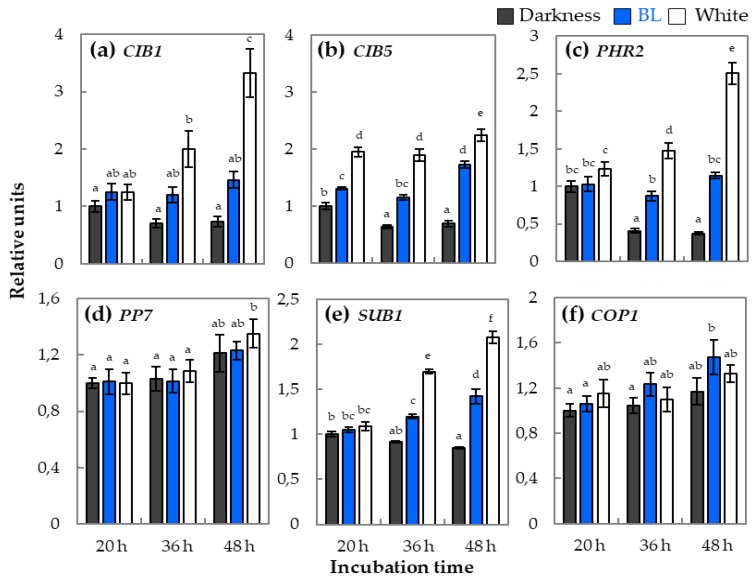
The analysis of relative expression of genes encoding regulatory proteins involved in light signaling, such as *CIB1* (**a**), *CIB5* (**b**), *PHR2* (**c**), *PP7* (**d**), *SUB1* (**e**), and *COP1* (**f**), in WT dormant *Arabidopsis* (Col-0) seeds incubated for 20, 36, and 48 h on water in different light conditions (BL and white light of intensity = 180 µmol m^−2^ s^−1^, darkness) at temperature of 25 °C: The transcript level was normalized to reference genes (*ACT7*, *APC2*, and *HBT*) and to the internal control, which was the expression value of each particular gene observed in seeds incubated for 20 h in darkness. Three biological and two technical replicates were performed. The bars show the relative units ± SD, letters a-f indicate homogeneous groups for *p* ≤ 0.05.

Due to the fact that in signaling pathway of BL are involved different regulatory proteins (i.e., CIB, PHR2 (PHOTOLYASE/BLUE LIGHT RECEPTOR 2), PP7 (SERINE/THREONINE PHOSPHATASE 7), SUB1 (ARABIDOPSIS CALCIUM-BINDING PROTEIN 1), and COP1), the expression profiles of genes coding them were investigated in samples obtained from germinating WT dormant seeds. Obtained results showed correlation between light conditions in which WT dormant seeds were incubated and expression of *CIB1* and *CIB5* genes (Figure 3a,b). In darkness, expressions of *CIB1* and *CIB5* were lower in comparison to that which was much higher in BL and white light conditions. Moreover, the presence of BL and white light increased *CIB1* and *CIB5* expression during incubation, while in darkness, the opposite effect was observed. In the case of *PHR2*, after 20 h of incubation of WT dormant seeds, its expression was at a similar level in BL and darkness and a bit higher in the presence of white light. However, within the duration of incubation, the expression of *PHR2* decreased in darkness, was unchanged in the presence of BL, and almost doubled in white light (Figure 3c). The *PP7* gene shows similar expression levels in all light conditions after 20 and 36 h of incubation (Figure 3d). At later time point of incubation (48 h), expression of *PP7* increased in all light conditions, with a tendency to be higher in samples incubated in white light. The expression of *SUB1* after 20 h of incubation was similar in samples isolated from all light conditions (Figure 3e). Within the duration of seed incubation, the level of *SUB1* transcripts in darkness slightly decreased but increased in the presence of BL and white light. In the case of *COP1* gene, its expression in darkness was lower in comparison to light conditions and did not changed after 20 h of germination. Interestingly, the presence of light, especially the BL wavelength, stimulated *COP1* expression after 36 and 48 h of incubation (Figure 3f).

**Figure 4:**
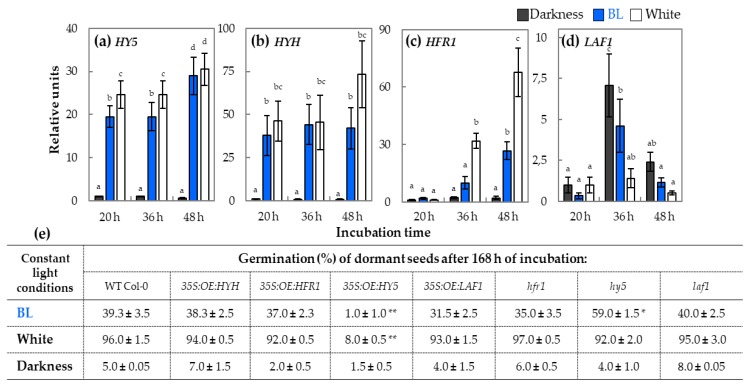
The analysis of the relative expressions of genes encoding transcription factors related to light signaling, such as *HY5* (**a**), *HYH* (**b**), *HFR1* (**c**), and *LAF1* (**d**), in samples isolated from WT dormant *Arabidopsis* (Col-0) seeds incubated on water for 20, 36, and 48 h in different light conditions (BL and white light of intensity = 180 µmol m^−2^ s^−1^, darkness) at temperature of 25 °C: The transcript level was normalized to reference genes (*ACT7*, *APC2*, and *HBT*) and to the internal control, which was the expression value of each particular gene observed in seeds incubated for 20 h in darkness. Three biological and two technical replicates were performed. The bars show the relative units ± SD, letters a-d indicate homogeneous groups for *p* ≤ 0.05. The germination % of dormant *Arabidopsis* seeds (in Col-0 background: WT Col-0, *35S:OE:HYH*, *35S:OE:HFR1*, *35S:OE:HY5*, *35S:OE:LAF1*, *hfr1*, *hy5*, and *laf1*) after 168 h of incubation on water in the presence of constant BL and white light (intensity = 180 µmol m^−2^ s^−1^) or in darkness at temperature of 25 °C (**e**). Experiments were conducted in three biological and two technical replicates (100 seeds per each replicate). Results are presented as mean ± SD, * and ** indicate the significant differences for *p* ≤ 0.05 and *p* ≤ 0.01 respectively.

The expression patterns of light-related transcription factors, such as *HY5*, *HYH*, *HFR1*, and *LAF1*, were also investigated. The expression of all these genes was strongly correlated with light conditions (Figure 4). The results of qRT-PCR analysis indicated that it was mostly the BL component of the light spectrum that stimulated not only *HY5* and *HYH* but also *HFR1* expression (Figure 4a–c). The expressions of all these 3 genes were at drastically low levels in seeds incubated in darkness. In the case of *LAF1*, the pattern was much different, as the highest expression was observed in seeds incubated in darkness, while the presence of white light kept it at rather low level (Figure 4d). The presence of BL had also a reducing effect on *LAF1* expression, but the result was not so strong as the one caused by white light.

The possible involvement of genes encoding light-related transcription factors in the mechanism of BL-mediated dormancy alleviation was verified also in germination assays of dormant *Arabidopsis* Col-0 seeds: WT and overexpression transformants, such as *35S:OE:HY5, 35S:OE:HYH, 35S:OE:HFR1*, and *35S:OE:LAF1*, and T-DNA insertion mutants, i.e., *hfr1*, *hy5*, and *laf1* (Figure 4e). All tested seeds did not germinate in darkness. The dormancy was alleviated by the constant presence of BL or white light (180 µmol m^−2^ s^−1^) in all tested types of seeds except *35S:OE:HY5*. After 168 h of incubation, approx. 30–40% and 90% of seeds WT Col–0, *35S:OE:HYH, 35S:OE:HFR1*, and *35S:OE:LAF1* were germinated respectively on BL and white light, while in the case of *35S:OE:HY5*, just 1–8% seeds germinated in these conditions (Figure 4e). In the case of T-DNA insertion mutants, the BL effect on dormancy alleviation was significantly stronger in the case of seeds of *hy5* mutant (Figure 4e).

### 2.3. The Analysis of Expression Profiles of Genes Involved in GA Metabolism and Signaling in Light-Dependent Germination of Dormant Seeds

**Figure 5:**
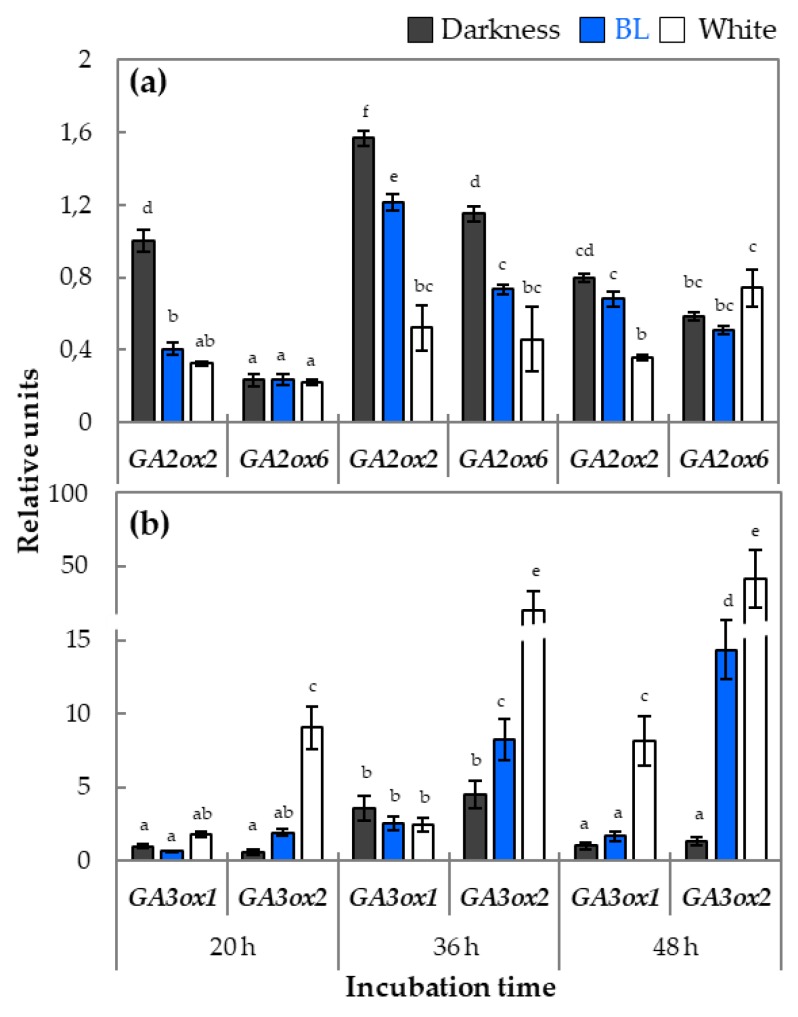
The relative expressions of genes encoding enzymes involved in GA metabolism: degradation—*GA2ox2* and *GA2ox6* (**a**)—and biosynthesis—*GA3ox1* and *GA3ox2* (**b**), in samples isolated from WT dormant *Arabidopsis* (Col-0) seeds incubated on water for 20, 36, and 48 h in different light conditions (BL and white light of intensity = 180 µmol m^−2^ s^−1^, darkness) at temperature of 25 °C. The transcript level was normalized to reference genes (*ACT7*, *APC2*, and *HBT*) and to the internal control, which was the expression value of *GA2ox2* (Figure 5a) or *GA3ox1* (Figure 5b) obtained in darkness after 20 h of incubation. Three biological and two technical replicates were performed. The bars show the relative units ± SD, letters a-f indicate homogeneous groups for *p* ≤ 0.05.

**Figure 6:**
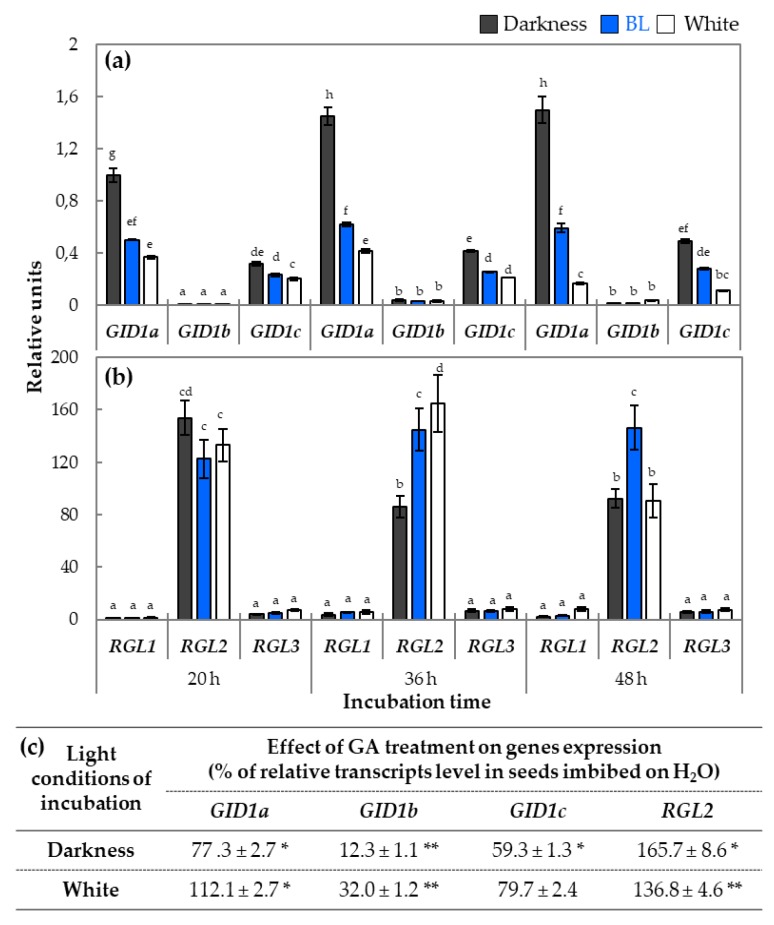
The relative expressions of genes encoding proteins involved in GA signaling, such as *GID1a*, *GID1b*, and *GID1c* (**a**) and *RGL1*, *RGL2*, and *RGL3* (**b**), in samples isolated from WT dormant *Arabidopsis* (Col-0) seeds incubated on water for 20, 36, and 48 h in different light conditions (BL and white light of intensity = 180 µmol m^−2^ s^−1^, darkness) at temperature of 25 °C: The transcript level was normalized to reference genes (*ACT7*, *APC2*, and *HBT*) and to the internal control, which was the expression value of *GID1a* (Figure 6a) or *RGL1* (Figure 6b) obtained in darkness after 20 h of incubation. Three biological and two technical replicates were performed. The bars show the relative units ± SD, letters a-h indicate homogeneous groups for p ≤ 0.05. The effect of GA (50 µM) treatment on *GID1a*, *GID1b*, *GID1c*, and *RGL2* expression in samples isolated from WT dormant *Arabidopsis* seeds incubated in darkness or white light for 20 h by qRT-PCR method (**c**). Results are expressed as % of relative transcript levels presented in seeds imbibed on water ± SD, * and ** indicate the significant differences for *p* ≤ 0.05 and *p* ≤ 0.01 respectively.

**Figure 7:**
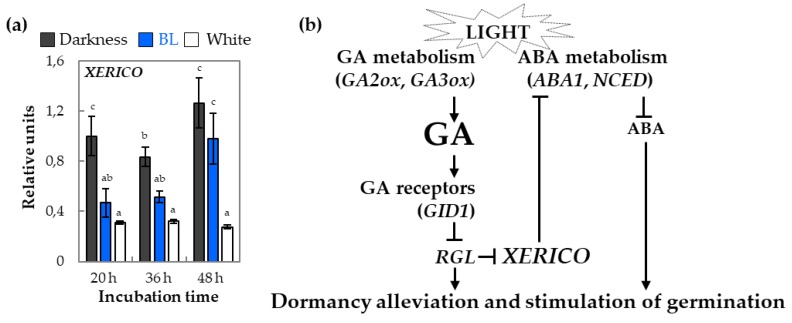
The relative expression of the *XERICO* gene in samples isolated from WT dormant *Arabidopsis* (Col-0) seeds incubated on water for 20, 36, and 48 h in different light conditions (BL and white light of intensity = 180 µmol m^−2^ s^−1^, darkness) at temperature of 25 °C (**a**): The transcript level was normalized to reference genes (*ACT7*, *APC2*, and *HBT*) and to the internal control, which was the expression value of *XERICO* gene obtained in darkness after 20 h of incubation. Three biological and two technical replicates were performed. The bars show the relative units ± SD, letters a-c indicate homogeneous groups for *p* ≤ 0.05. (**b**) The schematic presentation of the influence of light on GA and ABA metabolism and transduction of signal induced by these phytohormones during dormancy alleviation of imbibed seed: Light releases dormancy by modulation of expression of genes involved in GA and ABA metabolism, leading to changes in GA and ABA concentration and further in GA-dependent signal transduction via *GID1*, *RGL*, and *XERICO* genes.

The qRT-PCR analysis of relative expression of genes playing a role in GA metabolism and signaling was performed in WT dormant seeds incubated for 20, 36, and 48 h in different light conditions (darkness, BL, and white light) (Figure 5, Figure 6 and Figure 7).

The effect of light conditions on the expression of genes involved in GA degradation was investigated on the examples of two representatives from *GA2ox* group, such as *GA2ox2* and *GA2ox6* (Figure 5a). Interestingly, the expression profiles of both genes indicated that, in the presence of BL and white light, their transcript levels were lower in comparison to darkness. The differences in expression of *GA2ox2* was much higher especially after 20 h and 36 h and, in the case of *GA2ox6*, after 36 h (Figure 5a). Among the genes involved in GA biosynthesis, *GA3ox1*, *GA3ox2*, and *KAO2* were examined (Figure 5b, Figure S1). The expression profiles of both *GA3ox* genes showed correlation between light conditions of incubation and the transcript level in the case of *GA3ox2*, with a tendency that, in darkness, expression of *GA3ox2* was lower than in the presence of BL and white light (Figure 5b). The analysis of the *KAO2* relative expression indicated that its transcript level was higher in seeds incubated under white light for 20 h in comparison to BL and darkness and was equal in all light conditions after 36 h, while at the late time point of germination (48 h), the lack of light resulted in doubling its expression in comparison to the presence of BL or white light (Figure S1).

To assess in more details the impact of BL on the transcript abundance of genes related to GA signaling, the characteristic of expression profiles of genes encoding GA receptors, such as *GID1a*, *GID1b*, and *GID1c*, as well as three genes encoding DELLA factors *RGL1*, *RGL2*, and *RGL3* were performed in samples analogous as in the case of genes involved to light signaling and GA metabolism (Figure 6). The expression profile of *GID1a* and *GID1c* under white and BL was significantly lower than in darkness (Figure 6a). Interestingly, the expression profile of *GID1b* was much lower than that of the two other *GID1* genes.

The further analysis of the expression of three *RGL* genes indicated that the highest transcript level had *RGL2* (Figure 6b). Moreover, the expression of *RGL2* gene after 20 h of incubation was at a similar level in samples obtained from all light conditions, while after 36 h, it decreased in darkness and was lower by approx. 40% in comparison to BL and white light conditions. At the later investigated time point of germination (48 h), the transcript level of *RGL2* in samples incubated on white light decreased and obtained similar levels as the one observed in darkness, while it was still high in samples exposed to BL. The expressions of *RGL1* and *RGL3* did not show significant differences in all applied light conditions (Figure 6b).

To examine in more details the potential interaction between light and GA signaling pathways, additional qRT-PCR analyses of *RGL2* and all three *GID1* genes expressions were performed in samples obtained from germinating WT dormant seeds treated with GA solution (Figure 6c). It was observed that exogenous GA decreased relative *GID1b* and *GID1c* expression in darkness and in the presence of white light. Interestingly, the expression of *GID1a* decreased in GA-treated seeds incubated in darkness but increased in white light. In the case of *RGL2*, the application of GA resulted in an increase of *RGL2* expression for about 65 and 37%, respectively, in darkness and in white light (Figure 6c).

The potential interaction between GA and ABA metabolism and signaling pathways in light- dependent germination of WT dormant *Arabidopsis* seeds was verified by the qRT-PCR analysis of the transcript level of the *XERICO* gene (Figure 7). The obtained results indicated that, in germinating WT dormant seeds, the expression of this gene is negatively regulated by the presence of BL and white light.

### 2.4. The Expression Patterns of Genes Involved in ABA Metabolism and Signaling in Light-Dependent Germination of Dormant Seeds

**Figure 8:**
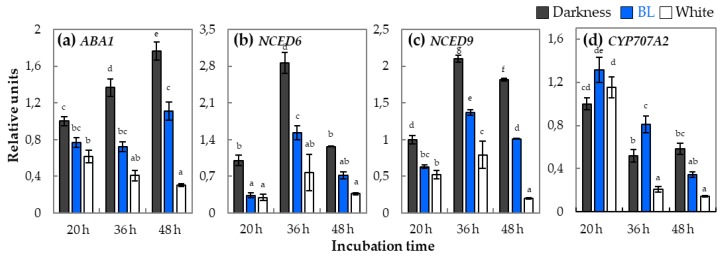
The analysis of relative expressions of genes encoding enzymes involved in ABA metabolism, such as *ABA1* (**a**), *NCED6* (**b**), *NCED9* (**c**), and *CYP707A2* (**d**), in samples isolated from WT dormant *Arabidopsis* (Col-0) seeds incubated on water for 20, 36, and 48 h in different light conditions (BL and white light of intensity = 180 µmol m^−2^ s^−1^, darkness) at temperature of 25 °C: The transcript level was normalized to reference genes (*ACT7*, *APC2*, and *HBT*) and to the internal control, which was the expression value of each particular gene observed in seeds incubated for 20 h in darkness. Three biological and two technical replicates were performed. The bars show the relative units ± SD, letters a-g indicate homogeneous groups for *p* ≤ 0.05.

For the better understanding of the mechanism of BL action during germination of WT dormant seeds, the expression patterns of genes involved in ABA biosynthesis, such as *ABA1, NCED6*, and *NCED9* (Figure 8a–c), and in degradation, such as *CYP707A2* (Figure 8d), were investigated. The transcript levels of *ABA1, NCED6*, and *NCED9* genes depend on the light conditions as they were the highest in seeds incubated in darkness and much lower in the presence of BL and white light. For the ABA degradation gene—*CYP707A2*—a clear pattern according to different light conditions was not observed. The relatively high expression of *CYP707A2* gradually decreased within the duration of incubation. After 48 h of imbibition *CYP707A2* expression was significantly higher in darkness in compare to BL and white light (Figure 8d).

**Figure 9:**
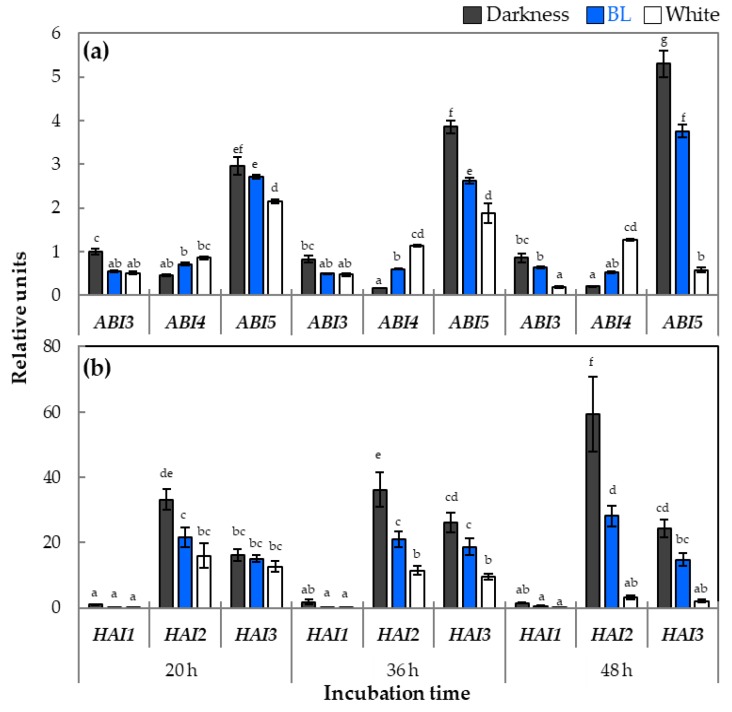
The relative expressions of genes encoding proteins involved in ABA signaling, such as *ABI3, ABI4*, and *ABI5* (**a**) and *HAI1, HAI2*, and *HAI3* (**b**), in samples isolated from WT dormant *Arabidopsis* (Col-0) seeds incubated on water for 20, 36, and 48 h in different light conditions (BL and white light of intensity = 180 µmol m^−2^ s^−1^, darkness) at temperature of 25 °C: The transcript level was normalized to reference genes (*ACT7*, *APC2*, and *HBT*) and to the internal control, which was the expression value of *ABI3* (Figure 9a) or *HAI1* (Figure 9b) obtained in darkness after 20 h of incubation. Three biological and two technical replicates were performed. The bars show the relative units ± SD, letters a-g indicate homogeneous groups for *p* ≤ 0.05.

To verify the impact of BL on the transcripts abundance of genes related to ABA signaling, the characteristic of expression profiles of *ABI* and *HAI* genes encoding, respectively, ABI transcription factors and protein phosphatases type 2C was performed (Figure 9).

The obtained results indicated that the *ABI3, ABI5, HAI1, HAI2*, and *HAI3* genes had significantly higher relative expressions in samples obtained from WT dormant seeds germinated in darkness than those incubated under BL and/or white light (Figure 9). Moreover, these differences increased within the duration of incubation. Interestingly, in the case of the *ABI4* gene, the reverses trend was observed, as the presence of BL and/or white light was positively correlated with its transcript level (Figure 9a).

## 3. Discussion

### 3.1. Blue Light Alleviates Dormancy and Stimulates Germination of Arabidopsis Seeds

The scientific literature provides us relatively much information about the role of R/FR light in seed germination of dicot plants, while the role of blue light (BL) is mainly discussed in the context of seed biology of monocot plants [15]. Studies conducted on mutants of *Arabidopsis* lacking active forms of R/FR light receptors phy confirmed that these proteins are responsible for the perception of light inducing seed germination and that R/FR light plays an important role in this process [30]. It is also known that, for monocot plants, such as barley (*Hordeum vulgare* L.) and wheat (*Triticum aestivum* L.), BL light received by cry inhibits germination of freshly harvested seeds of these species [28,31,32]. Surprisingly, the role and the mechanism of action of BL in seed biology of dicot plants including *Arabidopsis* is still very poorly understood. In this particular study, it was found that dormancy of freshly harvested *Arabidopsis* seeds which were unable to germinate in darkness (at 25 °C) can be released: 1) during dry after-ripening for at least 8 weeks or 2) by continuous or even short treatment of imbibed WT dormant seeds by BL and/or white light (Figure 1, Table S1). Worth noticing is that the effect of dormancy alleviation and germination stimulation mediated by BL and/or white light depends on the depth of dormancy. Further biological tests unrevealed also that incubation of WT dormant seeds in the presence of BL at range of intensities from 20 up to 180 µmol m^−2^ s^−1^ resulted in a similar stimulatory effect (Figure 1b). This result showed that both relatively low and high BL light intensity values stimulate germination; thus, in the natural environment its effect could be noticeable in fluctuating conditions of irradiance caused, i.e., by the presence of clouds and/or air pollutants. Based on these results, it is concluded that dormancy alleviation and stimulation of germination by BL most probably is a low fluence response (LFR) dependent on phyB. These findings are supported by earlier published results, where it was demonstrated that, during photoinduced germination of *Arabidopsis* seeds, phyA opposed to phyB mediates very low fluence response (VLFR) [7,17]. In this particular study, it was also demonstrated that pre-incubation of WT dormant *Arabidopsis* seeds in darkness for 24 h before 3 h illumination of them by BL decreased its stimulating effect, while the shorter pre-treatment in darkness for 6 h had an opposite effect (Suppl. Table 1). Interestingly, to break dormancy, it was required to apply BL for at least 24 h starting from the beginning of imbibition process or at a min. of 3 h of BL illumination but on seeds which were previously pre-incubated for 6–24 h in darkness (Table S1). These observations bring us to the conclusion that not only the duration of seed illumination by BL but also, more importantly, at which step of seed germination the BL is applied are crucial for the dormancy release. It seems that the sensitivity of WT dormant seeds to BL stimulus is changing within the duration of incubation, with the highest sensitivity occurring between 6 and 9 h of germination. Following this conclusion, it can be suggested that pre-imbibition of WT dormant seeds in the dark (especially between 6 and 24 h) do not allow for the removal of negative regulators and/or synthesis of stimulators of germination, leading then to inhibition of this process even in the presence of BL applied after 24 h of imbibition.

### 3.2. The phy Perceive Blue Light in Germinating Dormant Arabidopsis Seeds

The role of BL in germination of dicot seeds and its mechanism of action have not been yet well elucidated. It is still unclear which particular genes are involved in the BL signaling pathway and what target genes are responsible for regulation of germination of WT dormant *Arabidopsis* seeds. There are evidences that the perception of BL occurs mainly through cry, but it can also take place through proteins from phy family [33,34]. Therefore, to verify which of these photoreceptors might be involved in the perception of BL in germinating seeds, the qRT-PCR analysis of genes, such as *CRY1*, *CRY2*, *CRY3, PHYA*, and *PHYB* encoding these regulatory proteins, were performed. It was shown that *CRY1* and *CRY2* expression in WT dormant *Arabidopsis* seeds incubated in all tested light conditions was at a relatively similar level and did not depend on light conditions (Figure 2a). In the case of *CRY3* gene, its expression in germinating WT dormant seeds was much lower in compare to two other homologs and, furthermore, was positively correlated with the presence of white light (Figure 2a). In the later stages of germination (after 48 h), the relatively small stimulatory effect of BL on *CRY3* expression was also observed. The expression profile of *CRY3* suggests that this gene may play an important role in seed germination but that its biological function seems to be different from *CRY1* and *CRY2*. The cry3 protein, unlike cry1 and cry2, has a different type of photolyase activity and has the ability to bind to ssDNA (single-stranded DNA) and RNA and to repair some types of damages caused, e.g., by UV radiation [14,35]. In addition, unlike other cry, it is localized in chloroplasts and mitochondria; thus taking into account the biochemical activity of cry3, it may mean that it is involved in the protection of organellar genomes [14,36]. Therefore, it can be possible that during activation of the metabolism in germinating seeds, cry3 may be involved in protection of mitochondrial DNA against oxidative damages.

Considering the role of phy receptors in *Arabidopsis* seeds germination controlled by BL, it was discovered that only *PHYA* expression significantly decreased under BL and white light while *PHYB* was at a relatively low, stable level in each light condition (Figure 2b). This is in agreement with earlier research performed on *Arabidopsis* seedlings, which showed that *PHYA* expression was strongly inhibited by white light, and a smaller effect of light was observed on *PHYB* [7,37,38]. To decipher the potential correlation between cry (BL)- and phy (R/FR)- related pathways in light-mediated dormancy alleviation, the effect of FR on the stimulation of WT dormant *Arabidopsis* seeds germination by BL was verified by biological assay (Figure 2c). It was discovered that the application of FR after BL illumination during germination of dormant WT seeds significantly reduced the stimulatory effect of BL on this process. Moreover, the results of germination assay of dormant WT and mutants of genes encoding particular cry and phy indicated that the dormancy was alleviated by the constant presence of BL or white light (180 µmol m^−2^ s^−1^) in all tested types of seeds except *phyB* (Figure 2c,d). Based on that, it is proposed that, among cry and phy, the primary role in BL-mediated dormancy alleviation plays phyB, which by its own induced cascade of reactions (i.e., via interaction with PIL5) may lead to modulation of expression of downstream genes related to phytohormones metabolism and/or signaling. This assumption is supported by earlier studies, where it was demonstrated that light activates the degradation of PIL5 protein to promote germination through GA in *Arabidopsis* seeds [3,39]. Our research on BL-mediated dormancy alleviation of *Arabidopsis* seeds clearly shows that BL induces changes at the molecular level, altering transcription of specific genes, i.e., involved in light, GA/ABA metabolism, and/or signaling, and that phyB plays an important role in this process.

### 3.3. The *phy–cry*-Related Mechanism of Blue Light Action in Dormancy Alleviation and Germination of Arabidopsis Seeds Requires Interaction with Various Regulatory Proteins

The mechanism of cry action and its activity regulation in germinating seed has not been yet thoroughly understood. Up to date, it is known that, in plants under the influence of BL, cry undergo conformational changes, which allows them to interact with a number of specific protein regulators. To decipher in more details the role of genes coding proteins interacting with cry, the expression patterns of particular genes, such as *CIB1*, *CIB5*, *PHR2*, *PP7*, and *SUB1*, were investigated (Figure 3). The results of expression analysis of both *CIB* genes showed that the level of their transcripts in subsequent stages of germination of WT dormant seeds is stimulated by both BL and white light (Figure 3a,b). A different tendency was observed in the dark, where *CIB* expression gradually decreased during incubation (Figure 3a,b). The level of *CIB1* and *CIB5* transcripts in WT dormant seeds incubated under BL was intermediate between the level of expression in darkness and white light. These results suggest that both *CIB* genes may play an important role in transduction of BL-induced signal in germinating dormant seeds. Among the other genes involved in the transduction of the signal induced by BL, of which the expression was analyzed in this work, are *SUB1*, *PHR2*, and *PP7*. It is currently unknown whether and what biological functions these genes can play in regulation of dormancy alleviation and germination by BL. However, since they are associated with BL-related signaling, an attempt was made to characterize their role in BL-dependent germination of WT dormant *Arabidopsis* seeds. The regulation of BL-dependent photomorphogenesis by SUB1 probably relies on the inhibition of the accumulation of transcription factor HY5 [40]. The analysis of *SUB1* expression in WT dormant *Arabidopsis* seeds showed that, during germination in the presence of BL and white light, the level of *SUB1* transcripts was increasing while, in darkness, was downregulated (Figure 3e). The next investigated gene, *PHR2*, encodes the protein which has photolytic activity regulated by BL [41]. The expression of the *PHR2* gene in WT dormant seeds dependent on light conditions (Figure 3c). It was downregulated in darkness while kept at a stable, relatively high level in BL and upregulated by light (Figure 3c). The results of research carried out in this work suggests the possible *PHR2* participation in BL-mediated dormancy alleviation of WT dormant *Arabidopsis* seeds. Another gene involved in the signaling of BL analyzed in this work was *PP7*, encoding a protein with the function of serine–threonine phosphatase [42]. The results of the analysis of *PP7* expression in germinating WT dormant *Arabidopsis* seeds showed that the level of its expression was not modulated by light conditions but was rather characterized by a constant level and only slightly increased in the later hours of germination (Figure 3d). Based on these results, the role of the *PP7* gene in the regulation of light-dependent dormancy alleviation cannot be unequivocally determined.

The plant photoreceptors cry and phy, after absorbing light impulse, induce many protein–protein interactions, i.e., CUL4 E3 ubiquitin ligase with key targets, such as transcription factors (i.e., HY5, HYH, HFR1, and LAF1), resulting in changes in expression profiles of defined genes and therefore modulation of whole metabolism of plant organisms. Affinity purification of CUL4 from mammalian cells identified various possible substrate receptors, including COP1 known to repress light signaling by targeting photoreceptors and downstream transcription factors for ubiquitination and proteasomal degradation [43,44]. In consequence, the influence of light activated cry and phy on COP1 enables modulation of specific light-related gene expression. Surprisingly, there are no data in the scientific literature about the role of *COP1* gene in light-dependent germination of dormant dicot seeds. The results of qRT-PCR analysis presented in this particular study indicated that light, especially the BL wavelength, stimulated COP1 expression in germinating WT dormant *Arabidopsis* seeds, with the higher impact at the later phases of this process, just before radical protrusion through layers covering an embryo (Figure 3f). This finding is confusing in the context of previously published data by Deng et al. [45], who demonstrated that, in *Arabidopsis* seedlings, mRNA level of *COP1* was not dependent on light conditions and that the COP1 activity plays a more important role in regulation of this process. Taking into account that, in germinating WT dormant seeds, the light-dependent regulation of COP1 expression was observed, it is postulated that, at the time proceeding the shift from heterotrophic (germinating seed) into phototropic stage (the seedling growth), the mechanism may function differentially, as it require also changes in *COP1* transcript levels. It is also possible that COP1 activity in seeds is regulated by changing the location from nuclear to cytoplasmic according to the dark or light conditions or through regulation systems associated with cry1 and cry2, as is the case in photosynthetic organs [46,47]. Moreover, there are evidences that cry conformation changes and that their activation occurs only under the influence of BL, thanks to which they can inhibit the activity of attached COP1, while in the dark, COP1 not inactivated by cry can perform ubiquitination of cry, causing degradation of this photoreceptors [48]. These reactions can be an example of a possible two-way deactivation mechanism of COP1 and cry in response to the absence or presence of BL in germinating WT dormant *Arabidopsis* seeds.

### 3.4. HY5 Transcription Factor is the Most Probably Involved in Modulation of Blue Light-Mediated Dormancy Alleviation of Arabidopsis Seeds

**Figure 10:**
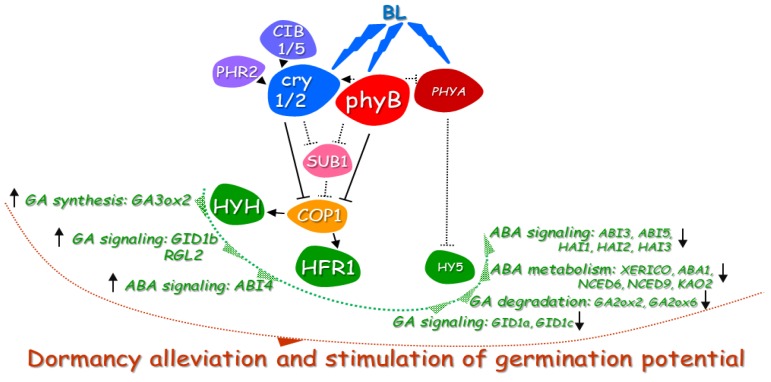
The hypothetical model of BL action in regulation of dormancy alleviation and germination potential of dormant *Arabidopsis* seeds: The BL downregulates the expression of *PHYA* but not *PHYB* neither *CRY1/2* photoreceptors. Decreased *PHYA* expression is associated with a possible low HY5 accumulation and/or its activity, leading to direct or indirect decrease in expression of genes encoding dormancy related genes. On the other hand, as the effect of BL illumination on the phyB and cry1/2 controlled by phyB, CIB1/5, and PHR2, the SUB1 and COP1 activity is suppressed, leading to HYH–-and HFR1-mediated activation of expression of specific genes involved in metabolism and signaling of GA and ABA, resulting in increase of germination potential. Dotted lines indicate hypothetical, possible interactions. Black arrows next to GA- and ABA-related genes indicate up- and downregulation of their expression.

In continuation of the elucidation of the role of particular elements of light signalling involved in control of dormancy alleviation and germination of WT dormant *Arabidopsis* seeds, the relative expression of genes coding transcription factors cooperating with cry and phy, such as *HY5*, *HYH*, *HFR1*, and *LAF1*, was investigated. It was proven that these transcription factors participate in COP1-dependent plant photomorphogenesis, regulating gene expression by binding to their G-box-containing promoter sequences [26,40]. The results presented in this particular study indicated that transcript levels of *HY5, HYH*, and *HFR1* in germinating WT dormant seeds depended on light availability (Figure 4a–c). The expression of these three genes in seeds incubated in darkness was very low, while under BL and white light, their transcript level was high. In addition, the transcript levels of *HY5*, *HYH*, and *HFR1* during seed incubation exposed to BL and white light were similar; therefore, it can be assumed that it is mainly BL light (as a part of white light spectrum) that induces their expression. Interestingly, in the case of *LAF1* gene, many different expression patterns were observed, with the tendency that the highest expression was in seeds incubated in darkness while the presence of BL and white light was correlated with its lower level (Figure 4d). The possible involvement of *HY5*, *HYH, HFR1*, and *LAF1* in the mechanism of BL-mediated dormancy alleviation was verified in germination assays of dormant seeds of *Arabidopsis* WT, T-DNA insertion mutants, and transformants characterized by overexpression of these genes (Figure 4e). It was observed that all these seeds did not germinate in darkness. Surprisingly, the constant presence of BL or white light (180 µmol m^−2^ s^−1^) alleviated dormancy in all tested types of seeds except *35S:OE:HY5*. Moreover, the BL effect on dormancy alleviation was significantly stronger in the case of seeds of *hy5* mutant (Figure 4e). It seems that an increase in *HY5* expression observed in samples isolated from WT dormant seeds exposed to BL is not correlated with HY5 protein accumulation and/or increase of its activity as seeds of *35S:OE:HY5* are unable to germinate in the presence of light (Figure 4a). Hence, the transcription factor encoded by *HY5* together with phyA pretend to be negative regulators of dormancy alleviation mediated by BL (but also white light based on the presented here in data), controlling transcript level of genes involved in ABA signaling (*ABI3*, *ABI5*, *HAI1*, *HAI2*, and *HAI3*), ABA metabolism (*XERICO*, *ABA1*, *NCED6*, *NCED9*, and *KAO2*), GA degradation (*GA2ox2* and *GA2ox6*), and GA signaling (*GID1a* and *GID1c*) (Figure 10). Opposed to phyA and HY5, phyB is recognized as positive regulator of BL-mediated dormancy release. Based on these results, it postulated that BL received by phyB alleviates dormancy of *Arabidopsis* seeds via *HY5*-dependent modulation of expression of downstream genes related to light, GA, and ABA, while biological functions of *HYH* and *HFR1* are associated with regulation of germination potential (Figure 10).

### 3.5. Effect of Blue Light on GA and ABA Metabolism in Germinating Dormant Seeds of Arabidopsis

The effect of R/FR light on germinability of dicot seeds such as *Arabidopsis* is usually related to changes in phytohormones (i.e., ABA and GA) content and/or signaling and generally associated with phytochromes activity, mostly phyA and phyB [19,20]. The stimulating effect of GA on *Arabidopsis* seed germination has long been shown, and the role of ABA in dormancy induction and maintenance has been also well documented by genetic and physiological studies [6,15]. In the case of BL, it is known that it inhibits germination of dormant grains and that this mechanism involves changes in gene expression leading to increase in ABA level and reduction of GA content and/or signaling [28]. The data presented in this study for the first time indicated that BL stimulates germination of freshly harvested WT dormant *Arabidopsis* seeds in the manner dependent on the depth of dormancy (Figure 1). Therefore, in subsequent studies, it was considered interesting to examine if the mechanism of BL action in regulation of WT dormant *Arabidopsis* seeds dormancy alleviation is due to modulation of metabolism and/or signaling of GA/ABA. From the literature, it is known that WT dormant *Arabidopsis* seeds are characterized by high level of ABA and that the their ability to synthesize GA is crucial for starting germination process [49,50]. The treatment of germinating WT dormant *Arabidopsis* seeds by ABA and PAC (inhibitor of GA synthesis) solutions in the presence of BL or darkness indicated that the BL effect on seed germination was strongly inhibited by the presence of both substances (Table 1). These results allowed us to conclude that the mechanism of action of BL involves the regulation of GA/ABA metabolism and/or their signaling pathways.

To further elucidate the stated above hypothesis, the expression profiles of genes related to GA and ABA metabolism were characterized. Three important genes involved in biosynthesis of GA level in germinating seeds are *GA3ox1*, *GA3ox2*, and *KAO2* [3,19]. In this study is shown that BL increased the level of transcript *GA3ox2* while did not significantly influence *GA3ox1* expression in comparison to the effect observed in darkness (Figure 5b). The expression of *KAO2* gene was at a similar level in WT dormant seeds exposed to all light conditions, with an exception of a much higher *KAO2* expression in WT dormant seeds incubated in darkness for 48 h (Figure S1). Based on that, it is postulated that possible altered GA content in WT dormant seeds germinating the presence of BL may partially result from increased expression of *GA3ox2* and not *GA3ox1* and *KAO2.* Due to the fact that, in germinating seeds, the content of GA is the result of biosynthesis and degradation processes, the expression of genes belonging to *GA2ox* was also investigated (Figure 5a). It was observed that, after 20 h of incubation, the transcript level of *GA2ox2* was much lower under BL and white light in comparison to darkness. Although this pattern changed during later steps of germination, it is possible that especially in early stages of germination, light-dependent inhibition of GA degradation is important for induction of BL-mediated dormancy alleviation and germination processes.

The investigation of expression of genes involved in ABA metabolism showed that transcript levels of *ABA1, NCED6*, and *NCED9* genes depend on the light conditions as it was much higher in WT dormant seeds incubated in darkness and much lower in the presence of BL and white light (Figure 8a–c). In the case of *CYP707A2*, it is also possible that the decrease in transcript level of this gene is more related to duration of incubation than to light conditions. It is known that some of these genes such as *NCED6* and catabolism-like *CYP707A2* are known to be regulated mostly by phy so its expression is related to R/FR light signaling [19]. On the other hand, there are evidences that, in dormant barley grains, BL perceived mostly by cry1 induced the expression of *NCED9* and limited expression of *ABA8* [28]. These findings support the assumption that BL may be one of the most important environmental factors that suppress dormant grains germination while in freshly harvested seeds of *Arabidopsis* this mechanism occurs in the opposite manner. All taken together, they support the statement that the BL-mediated dormancy alleviation of WT dormant seeds is related also to regulation of expression of genes involved in ABA metabolism.

The research studies aiming to decipher the mechanisms of regulation of GA and ABA metabolism have proved that these hormones can mutually regulate their own balance in germinating seeds. One of the most interesting gene involved in this kind of regulation is *XERICO*, a positive regulator of ABA synthesis that itself is negatively regulated by GA in DELLA-dependent manner [51]. Our results show that *XERICO* expression is related to light conditions and that, in germinating, WT dormant seeds is significantly lower in BL and white light conditions (Figure 7). This may suggest that *XERICO* is one of the genes involved in the BL-mediated dormancy alleviation and seed germination. Moreover, it indirectly shows that, under BL, there are possibly more GA which decrease *XERICO* expression under BL compared to darkness.

### 3.6. Effect of Blue Light on GA and ABA Signaling in Germinating Dormant Seeds of Arabidopsis

The mechanisms controlling light-dependent dicot seed germination include modulation of signaling pathways induced by phytohormones. This comprises changes in expression of GA and ABA signaling genes and/or proteins activity [3]. The regulatory proteins important for control of seed germination are GID1 receptors of GA that are responsible for GA-dependent degradation of other important signaling proteins—DELLAs [21,52]. Therefore, it was tempting to verify if BL may modulate expression of three genes belonging to *GID* family and, in consequence, alter GA signaling pathway during germination of WT dormant *Arabidopsis* seeds. Obtained results indicated that expression of *GID1a* and *GID1c* was downregulated by BL (Figure 6a). It is not surprising as there are evidences demonstrating that GA regulates *GID1a* expression through negative feedback loop [53]. In the case of *RGL* genes encoding DELLA proteins, the relative expression of *RGL1* and *RGL3* was much lower than *RGL*, and not modified in the presence of BL. These data may suggest that *RGL1* and *RGL3* are not involved in BL-dependent germination of WT dormant seeds. It is known that RGL1 and RGL3 are negative regulators of seed germination. Interestingly, the expression profile of *RGL2* indicated that its transcript level was maintained at a high level in the presence of BL at the early and later stages of germination (Figure 6b). These data may expand existing knowledge about the role of RGL2, which is actually recognized as a negative regulator in R/FR-dependent germination of *Arabidopsis*. It is possible that, in the presence of BL, the protein encoded by *RGL2* plays a different role. To decipher in more detail potential interactions between GA and light related pathways in WT dormant germinating seeds, the effect of GA treatment on expression of genes involved in this phytohormone signaling was determined (Figure 6c). Interestingly, the GA treatment of WT dormant seeds incubated in darkness resulted in a decrease of all *GID* transcripts while had a stimulatory effect on *RGL2.* On the other hand, the effect of GA application in the presence of white light resulted in an increase in *GID1a* and *RGL2* expression, while in darkness *GID1b* and *GID1c* were downregulated, but the negative effect was not as strong as that observed in darkness (Figure 6c). It is also worth to notice that, for all tested genes involved in metabolism and signal transduction of GA, the changes in expression caused by BL and white light were very similar. This may suggest that signal perception and transduction induced by BL and white light leading to these changes have identical molecular and biochemical background. Based on this information, we can presume that the mechanism of dormancy alleviation and stimulation of germination of WT dormant *Arabidopsis* seeds by BL includes regulation of GA perception and signal transduction due to the modulation of transcripts of *GID* and *RGL2* genes.

The studies on the role of BL light in modulation of ABA signaling during WT dormant *Arabidopsis* seeds germination revealed that expression of genes involved in this pathway depends on light conditions and that this specific patterns of expression can be responsible for decrease in ABA sensitivity and/or increase of GA content in WT dormant seeds germinating under BL (Figure 9). Among transcription factors from *ABI* family, we found two, *ABI3* and *ABI5*, to be downregulated by BL and white light (Figure 9a). Surprisingly, the opposite pattern was observed in the case of *ABI4*, as its transcript level was downregulated in darkness. With regards to ABA, it was demonstrated that its signaling in germinating *Arabidopsis* seeds depends on light conditions, i.e., R light, can activate pathways leading to destabilization or degradation of ABA signaling factors such as ABI3 or ABI5 (ABA Insensitive 3 or 5), in consequence resulting in inhibition of ABA signaling and stimulation of germination process [54,55,56]. The protein encoded by *ABI4* is known to be involved in ABA and GA crosstalk [57]. Upregulation of *ABI4* was previously observed, but it was correlated with increase of *CYP707A2* and two DELLA genes (*RGL1* and *RGL3*) and was phyA dependent [57]. Our data suggest that different *ABI* genes can play different roles during BL light-dependent seed germination and that these issues need to be further investigated. The signaling pathway of ABA includes also a protein phosphatase 2C, encoded by three *HAI* genes. Previous studies clearly indicate that products of *HAI* genes are important components (negative regulators) of ABA signaling pathway and interact with ABI [57,58]. The analysis made by us shows that expression of all *HAI* is light dependent (Figure 9b). The significantly lower expression of *HAI* genes was observed in seeds germinating under BL and white light, compared to darkness. Taking together all these results characterizing expression of ABA signaling genes, it was concluded that *HAI* genes may have significant influence on modulation of ABA sensitivity in seeds germinating in these conditions. Therefore, we can summarize that mediated by BL dormancy alleviation and stimulation of WT dormant *Arabidopsis* seeds, germination is related not only to changes in expression of genes related to GA and ABA metabolism but also to their signaling pathways.

## 4. Materials and Methods

### 4.1. Plant Material and Germination Assays

*Arabidopsis thaliana* ecotype Columbia (Col-0) wild type (WT) and homozygous lines of transformants characterized by overexpression of a specified gene (*HY5*, At5g11260; *HYH*, At3g17609; *HFR1*, At1g02340; and *LAF1*, At4g25560), T–DNA insertion single mutants (*phyA*, salk_020360; *phyB*, salk_022035; *cry1***,** salk_042397*; cry3*, salk_085913; *hfr1*, salk_049497; *hy5* salk_056405; and *laf1*, salk_079609), as well as double mutant of *cry1cry2* in background Ler-0 were used in this study. The WT and insertion single mutant seeds were purchased from NASC (The Nottingham Arabidopsis Stock Center, http://arabidopsis.info/), transformants lines were generated and selected as part of this research according to the protocol described below. The double mutant of *cry1cry2* was provided by Margaret Ahmad and previously described by Ahmad et al. [59]. The T-DNA mutants were genotyped according to the protocol provided by NASC at http://signal.salk.edu/. The homozygous lines *35S:OE:HY5*, *35S:OE:HYH*, *35S:OE:HFR1*, and *35S:OE:LAF1* were selected and verified as described in Section 4.2. In all qRT-PCR analyses as well as in most of genetic and biological studies seeds in *Arabidopsis* Col-0 ecotype were used. The only exception was the germination assay of mutants of genes encoding particular photoreceptors, where additionally to WT Col-0 and *phyA*, *phyB*, and *cry1, cry3* in the Col-0 ecotype and WT Ler-0 and *cry1cry2* in the Ler–0 ecotype were used (results presented in Figure 2d).

Seeds were propagated from plants growth in soil mixed with perlit (4:1, v:v) in controlled conditions in a phytochamber: long day photoperiod (16 h of light and 8 h of darkness) at 22 °C. Freshly harvested seeds were divided in two parts: one was stored in darkness at −30 °C to prevent dormancy alleviation (dormant seeds), and the other one was stored in the dark at 22 °C for at least 8 weeks to allow dormancy release (afterripened, nondormant seeds).

Seeds characterized by different depths of dormancy were sown on Petri dishes (100 seeds per plate, two technical repetitions x three independent biological replicates) filled with two layers of filter paper (EUROCHEM BGD, Tarnow, Poland) in the presence of 2.7 mL of sterile MiliQ water and/or appropriate solution of chemicals, such as ABA (SIGMA–ALDRICH, MI, USA) and/or PAC—an inhibitor of GA synthesis (SIGMA–ALDRICH) at constant temperature of 25 °C. To avoid pathogen infections, the PPM (Plant Preservative Mixture, Plant Cell Technology, Washington, DC, USA) was added to each germination medium (0.01%; *v/v*). Seeds were germinated under different light conditions: white light (intensity = 180 µmol m^−2^ s^−1^, source: lamps PANASONIC FL40SS), BL (range of intensities = 20–180 µmol m^−2^ s^−1^, source: LED SL 3500 lamp, Photon System Instruments, Brno, Czech Republic), or darkness. The BL was emitted with optimum wavelength of 447 nm. For checking if BL effect can be reversed by FR, impulse seeds were treated with impulse of FR light (60 µmol m^−2^ s^−1^, λ 735 nm) supplied from LED SL 3500 lamp (Photon System Instruments, Brno, Czech Republic) for 5 min, after 12 h of imbibition under BL, seeds were transferred under BL immediately after sowing.

Protrusion of the endosperm and seed coat by elongated radicle was considered as the end of germination.

### 4.2. Construct Preparation, Plant Transformation, and Phenotypic Analysis of Transgenic Plants

The specific primers were designed to amplify coding sequences of 4 genes of transcription factors, such as *HY5* (L: CACCATGCAGGAACAAGCGACTA, R: TCAAAGGCTTGCATCAGCAT), *HFR1* (L: CACCATGTCGAATAATCAAGCTTTC, R: TCATAGTCTTCTCATCGCA), *HYH* (L: CACCATGTCTCTCCAACGACCCA, R: TTAGTGATTGTCATCAGTTTTAGG), and *LAF1* (L: CACCATGGCGAAGACGAAATATGGA, R: TTACGTCGTTGTTGATGGAGAA). All coding sequences of the indicated above genes were fused behind the cauliflower mosaic virus 35S promoter of the *Agrobacterium tumefaciens* using pENTR^TM^/D–TOPO (Invitrogen, Waltham, MA, USA) and pK7WG2D,1 (Invitrogen) vectors by Gateway^TM^ LR Clonase^TM^ II Enzyme MIX kit (Invitrogen). The plasmid constructs were mobilized into the *Agrobacterium* strain EHA and transformed into WT *Arabidopsis* Col-0 plants by the floral dip method, as described by Clough and Bent [60]. After that step, T1 seeds were bulk collected and plated on Murashige and Skoog (MS) medium (SIGMA-ALDRICH), supplemented with 1% sucrose and 75 mg/mL kanamycin. Antibiotic-resistant F1 plants were identified after 10–14 days in a growth phytochamber. Then, they were transferred to a soil mixed with perlit (4:1, *v*:*v*) and allowed to grow and self-pollinate. The T2 progeny of individual T1 plants were harvested and analyzed further. At least 100 seeds of transgenic T2 and T3 lines were sown on MS plates containing 1% sucrose and 75 mg/mL kanamycin, cold treated at 8 °C for 4 days, and allowed to germinate by 4 days under constant white light (intensity = 180 µmol m^−2^ s^−1^) at 22 °C before being transferred to the long day photoperiod (16 h of light and 8 h of darkness) at the same temperature conditions.

The levels of expression of *HY5*, *HYH*, *HFR1*, and *LAF1* in corresponding to them obtained homozygous lines of transformants. T-DNA insertion mutants *hfr1*, *hy5*, and *laf1* were verified by qRT-PCR method using primers characterised in Table S2, and the results are presented in the Figures S2 and S3.

### 4.3. RNA Extraction and cDNA Synthesis

The samples of RNA used further for cDNA synthesis and qRT-PCR analysis of genes expressions were extracted from WT dormant *Arabidopsis* seeds imbibed for 20, 36, or 48 hours under constant light conditions: darkness, BL (180 µmol m^−2^ s^−1^), and white light (180 µmol m^−2^ s^−1^). Collected seeds were immediately frozen in liquid nitrogen and grinded using a tissuelyser (Precellys 24, Bertin Technologies, Montigny-le-Bretonneux, France). The total RNA was extracted using the CTAB method described previously by Chang et al. [61] and Cadman et al. [62]. Before cDNA synthesis, extracted RNA was treated with DNase I (SIGMA–ALDRICH) to eliminate any potential residues of plant genomic DNA. The cDNA was synthetized from 1.8 µg of total RNA of each biological sample, using Revert Aid H Minus First Strand cDNA Synthesis Kit (Thermo Fisher Scientific, Waltham, MA, USA) according to the manufacturer’s protocol in a thermocycler NEXUS (Eppendorf, Hamburg, Germany). The concentration and purity of RNA was validated on Nanodrop 2000 (Thermo Fisher Scientific), while RNA integrity was checked by the electrophoresis in 1% agarose gel.

### 4.4. Quantitative Real Time–PCR Analysis

The quantitative Real Time–PCR analysis was performed using SybrGreen Master Mix (ROCHE, Basel, Switzerland) and 200 ng of cDNA as a template. Reactions were run on LightCycler’ 96 (ROCHE, Basel, Switzerland). The relative expressions of following genes were analysed: 1) light signaling related genes: *PHYA* (At1g09570), *PHYB* (At2g18790), *CRY1* (At4g08920), *CRY2* (At1g04400), *CRY3* (At5g24850), *HY5* (At5g11260), *HYH* (At3g17609), *HFR1* (At1g02340), *LAF1* (At4g25560), *CIB1* (At4g34530), *CIB5* (At1g26260), *SUB1* (At4g08810), *PP7* (At5g63870), *PHR2* (At2g47590), and *COP1* (At2g32950); 2) genes involved in ABA metabolism and signalling: *ABA1* (At5g67030), *NCED6* (At3g24220), *NCED9* (At1g78390), *CYP707A2* (At2g29090), *ABI3* (At3G24650), *ABI4* (At2G40220), *ABI5* (At2G36270), *HAI1* (At5g59220), *HAI2* (At1g07430), and *HAI3* (At2g29380); and 3) genes involved in GA metabolism and signalling: *GA3ox1* (At1g15550), *GA3ox2* (At1g80340), *GA2ox6* (At1g02400), *GA2ox2* (At1g30040), *GID1a* (At3g05120), *GID1b* (At3g63010), *GID1c* (At5g27320), *RGL1* (At1g66350), *RGL2* (At3g03450), *RGL3* (At5g17490), *XERICO* (At2g04240), and *KAO2* (At2g32440). The characteristic of gene-specific primers used in qRT-PCR is shown in Table S2. The primers were designed using the Primer3 program (http://www.bioinformatics.nl/cgibin/primer3plus/primer3plus.cgi). The coding nucleotide sequences of investigated genes was taken from the NCBI database (http://www.ncbi.nlm.nih.gov/). In addition, analysis of homologous sequences was performed using the ClustalW program (http://www.ebi.ac.uk/Tools/msa/clustalw2/), and primer pairs were designed that were specifically bound to the sequence of the selected gene. The obtained results were calculated using tree reference genes: *ACT7* (*ACTIN7*, At5g09810), *APC2* (*ANAPHASE PROMOTING COMPLEX/CYCLOSOME 2*, At2g04660), and *HBT* (*HOBBIT*, At2g20000), described as genes with stable expression in germinating *Arabidopsis* seeds [63]. The values of relative expression obtained for each analysed gene in samples obtained from seeds after 20 h of imbibition in darkness was recognized as 1. On the graphs, the relative expression of genes is shown.

For studying the impact of GA on different *GID1* and *RGL2* genes, dormant *Arabidopsis* Col-0 seeds were treated for 20 h with GA in concentration of 50 µM at constant temperature of 25 °C in darkness or under white light (intensity = 180 µmol m^−2^ s^−1^). The results were expressed as % of relative transcript levels presented in seeds imbibed on water ± SD.

### 4.5. Statystical Analysis

The obtained results were analyzed using Statistica 13.1 Software (Statsoft, Cracow, Poland). The mean values were calculated, and statistically significant differences were evaluated using ANOVA analysis followed by Tukey’s HSD post hoc test for germination assays (* p ≤ 0.05; ** p ≤ 0.01) and qRT-PCR analysis of GA treated WT dormant Arabidopsis (Col-0) seeds (p ≤ 0.05). The ANOVA analysis and Duncan’s post hoc test were used to determine homogeneous groups in qRT-PCR analysis of WT dormant Arabidopsis (Col-0) seeds incubated on water for 20, 36, and 48 h in different light conditions (BL and white light and darkness) (p ≤ 0.05). Standard deviation (± SD) was also provided to indicate the variations associated with the particular mean values.

## 5. Conclusions

Based on obtained data, the hypothetical model of possible BL mechanism of action in regulation of dormancy alleviation and germination potential of *Arabidopsis* seeds is proposed (Figure 10).

It is proposed that the key photoreceptors involved in dormancy alleviation mechanism mediated by BL are phyB (Figure 10). The phyB by its own induced cascade of reactions but also by influencing cry1/2 activities may suppress the SUB1 and COP1 actions to modulate expression of genes encoding transcription factors, such as *HYH* and *HFR1*, leading to increase in transcript levels of downstream genes involved in GA synthesis (*GA3ox2*), GA signaling (*GID1b* and *RGL2*), and ABA signaling (*ABI4*). Besides of the phyB influence on cry1/2 action, CIB1/5 and PHR2 proteins may also have a positive effect on cry1/2 activities as the expressions of genes *CIB1/5* and *PHR2* are upregulated by BL. On the other hand, as the effect of BL-induced signal, the expression of *PHYA* decreases, which together with the possible inhibitory effect of phyB on phyA activity results in increase of *HY5* expression but not HY5 protein accumulation and/or increase of its activity. The transcription factor encoded by *HY5* seems to be a negative regulator of dormancy alleviation and germination mediated by BL (but also white light based on the presented data), controlling transcript level of genes involved in ABA signaling (*ABI3*, *ABI5*, *HAI1*, *HAI2*, and *HAI3*), ABA metabolism (*XERICO*, *ABA1*, *NCED6*, *NCED9*, and *KAO2*), GA degradation (*GA2ox2* and *GA2ox6*), and GA signaling (*GID1a* and *GID1c*). All taken together result in dormancy alleviation by BL and increase in germination potential of *Arabidopsis* seeds (Figure 10).

## Figures and Tables

**Table 1:** The effect of PAC (1 μM) and ABA (1 μM) solutions on germination rate of WT dormant *Arabidopsis* (Col-0) seeds incubated in darkness or under BL (180 µmol m^−2^ s^−1^) in constant temperature of 25 °C: Results were presented as % of germinated WT dormant seeds on water or on particular treatment ± SD after 48 and 72 h of incubation in constant temperature of 25 °C. Experiments were conducted in three biological and two technical replicates (100 seeds per each replicate), * and ** indicate the significant differences for *p* ≤ 0.05 and *p* ≤ 0.01 respectively.

Constant Light Conditions	Treatment	Germination (%) of WT Dormant Seeds after Time of Incubation	
		48 h	72 h
BL	H_2_O	14.33 ± 0.5	44.4 ± 2.5
	PAC (1 µM)	12.0 ± 0.5	15.0 ± 1.5 *
	ABA (1 µM)	3.0 ± 0.33 *	5.0 ± 0.33 **
Darkness	H_2_O	0.7 ± 0.5	0.7 ± 0.5
	PAC (1 µM)	2.0 ± 0.12	2.0 ± 0.5
	ABA (1 µM)	0.0 ± 0.0	1.0 ± 0.33

## Supplementary Materials

Supplementary materials can be found at https://www.mdpi.com/1422-0067/20/23/5882/s1.

## Abbreviations

ABA	Abscisic Acid
*ABA1*	*ARABIDOPSIS THALIANA ABA DEFICIENT 1*
*ABI3, 4* and *5*	*ABA INSENSITIVE3*, *4* and *5*
*ACT7*	*ACTIN 7*
*APC2*	*ANAPHASE PROMOTING COMPLEX* 2
BL	Blue Light
*CIB1* and *5*	*CRYPTOCHROME INTERACTING BASIC HELIX-LOOP-HELIX 1* and *5*
*CRY1, 2* and *3*	*CRYPTOCHROME 1*, *2*, and *3*
*COP1*	*CONSTITUTIVE PHOTOMORPHOGENIC 1*
CUL4	CULLIN4
*CYP707A2*	*CYTOCHROME P450, FAMILY 707, SUBFAMILY A, POLYPEPTIDE 2*
DELLA	DELLA factors encoded by *RGL* genes
FKF1	FLAVIN-BINDING, KELCH REPEAT, F-BOX 1
FR	Far Red Light
*FT*	*FLOWERING LOCUS*
GA	Gibberellins
*GA3ox1* and *2*	*GA 3–OXIDASE 1* and *2*
*GA2ox2* and *6*	*GA 2–OXIDASE 2* and *6*
*GID1a, 1b* and *1c*	GA INSENSITIVE DWARF *1a*, *1b*, and *1c*
*HAI1*, *2* and *3*	*HIGHLY ABA*–*INDUCED PP2C 1*, *2*, and *3*
*HBT*	*HOBBIT*
*HFR1*	*LONG HYPOCOTYL IN FAR* *-RED 1*
*HY5*	*ELONGATED HYPOCOTYL 5*
*HYH*	*HY5* *–HOMOLOG*
*KAO2*	*ENT* *–KAURENOIC ACID OXYDASE 2*
*LAF1*	*LONG AFTER FAR* *-RED LIGHT 1*
LFR	Low Fluence Response
LKP2	LOV KELCH PROTEIN 2
LLP BL	LOV/LOV domain-containing protein
LOV	Light, Oxygen, or Voltage photosensory domain
*NCED6* and *9*	*NINE*–*CIS EPOXYCAROTENOID DIOXYGENASE 6* and *9*
ND	Nondormant
PAC	Paclobutrazol
phot1 and 2	phototropin 1 and 2
*PHR2*	*PHOTOLYASE/BLUE LIGHT RECEPTOR 2*
*PHYA* and *B*	*PHYTOCHROMEA* and *B*
PIF	PHYTOCHROME INTERACTING FACTOR
PIL5	PIF3-LIKE 5
*PP7*	*Serine/Threonine Phosphatase 7*
PP2C	PROTEIN PHOSPHATASE TYPE 2C
PYR/PYL/RCAR	PYRABACTIN RESISTANCE/PYR1-LIKE/REGULATORY COMPONENT OF ABA RECEPTOR
R	Red Light
*RGL1*, *2* and *3*	*REPRESSOR OF GA*-*LIKE 1*, *2*, and *3*
SnRK2	SNF1-related protein kinases 2
SPA	SUPRESS PHYTOCHROME A-105
*SUB1*	*CALCIUM ION-BINDING PROTEIN SUB1*
VLFR	Very Low Fluence Response
WT	Wild Type
*XERICO*	gene encoding RING/U-box superfamily protein XERICO
ZTL	ZEITLUPE
