# Supplementary material for: phyB and HY5 are Involved in the Blue Light-Mediated Alleviation of Dormancy of Arabidopsis Seeds Possibly via the Modulation of Expression of Genes Related to Light, GA, and ABA
Source: Int J Mol Sci. 2019 Nov 23;20(23):5882. doi: 10.3390/ijms20235882 (PMC6928806; doi:10.3390/ijms20235882)
Supplement: Supplementary file 1 [file ijms-20-05882-s001.pdf]

International Journal of *Molecular Sciences*

MDPI

Supplementary Materials: Supplementary materials can be found at www.mdpi.com/xxx/s1.

phyB and HY5 are Involved in the Blue Light-Mediated Alleviation of Dormancy of *Arabidopsis* Seeds Possibly via the Modulation of Expression of Genes Related to Light, GA, and ABA

Marlena Stawska and Krystyna Oracz *

[1] Department of Plant Physiology, Institute of Biology, Warsaw University of Life Sciences – SGGW, Nowoursynowska str. 159, 02-776, Warsaw, Poland; krystyna_oracz@sggw.pl

* Correspondence: krystyna_oracz@sggw.pl; Tel.: (+48 22 59 325 35) (K.O.)

Supplementary Table S1. Effect of different combinations of pre– or post–incubations in darkness on BL light effect on germination rates of WT dormant *Arabidopsis* Col–0 seeds. Results are expressed as % of germinated seeds after 48 and 72 h of incubation in specific light conditions ± SD. Experiments were conducted in three biological and two technical replicates (100 seeds per each replicate).

Light conditions of incubation	Germination (%) of WT dormant seeds after time of incubation:	
	48 h	72 h
3 h BL > Darkness (const.)	3.0 ± 1.0 **	9.0 ± 1.0 **
6 h BL > Darkness (const.)	5.6 ± 0.5 **	12.0 ± 1.5 **
24 h BL > Darkness (const.)	26.5 ± 0.5 **	54.0 ± 2.0
48 h BL > Darkness (const.)	19.5 ± 2.5 *	50.6 ± 2.5
6 h Darkness > 3 h BL > Darkness (const.)	25.5 ± 1.0 *	46.6 ± 0.5
24 h Darkness > 3 h BL > Darkness (const.)	11.0 ± 0.5	27.0 ± 0.5 **
BL (const.)	14.3 ± 0.5	44.4 ± 2.5
Darkness (const.)	0.70 ± 0.5 **	0.7 ± 0.5 **

Supplementary Figure S1.

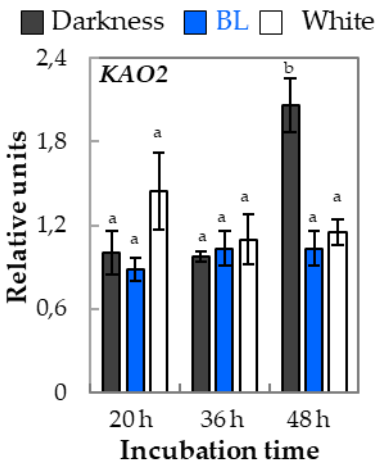

Supplementary Figure S1. The relative expression of *KAO2* gene in samples isolated from WT dormant *Arabidopsis* (Col–0) seeds incubated on water for 20, 36 and 48 h, in different light conditions: darkness, BL and white light, at temp. 25 °C. The transcripts level was normalized to reference genes (*ACT7*, *APC2*, *HBT*) and to the internal control which was the expression value of *KAO2* gene obtained in darkness, after 20 h of incubation. Three biological and two technical replicates were performed. The bars show the relative units ± SD.

Supplementary Figure S2.

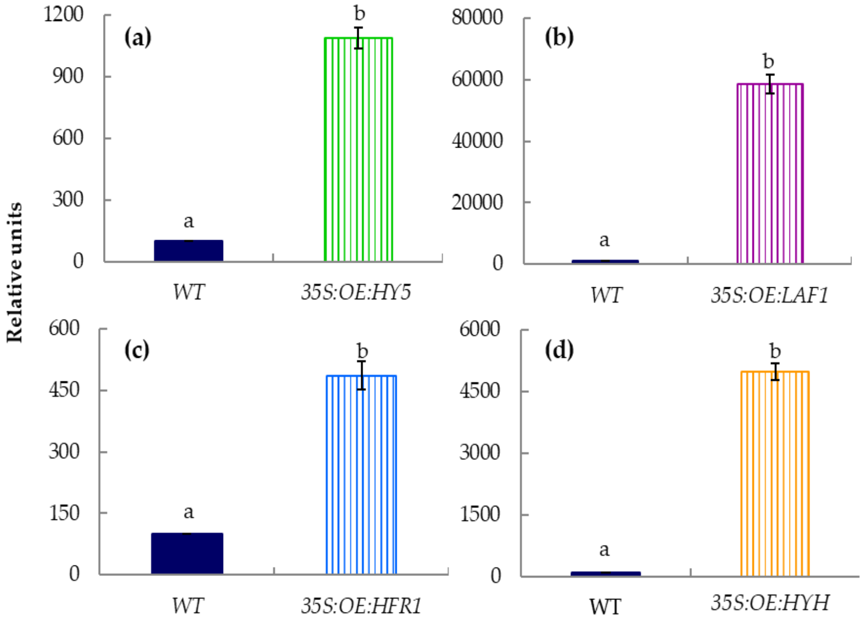

Supplementary Figure 2. The qRT-PCR analysis of relative expression of defined genes in 3 weeks old seedlings of overexpression lines of *Arabidopsis* transformants.

Supplementary Figure S3.

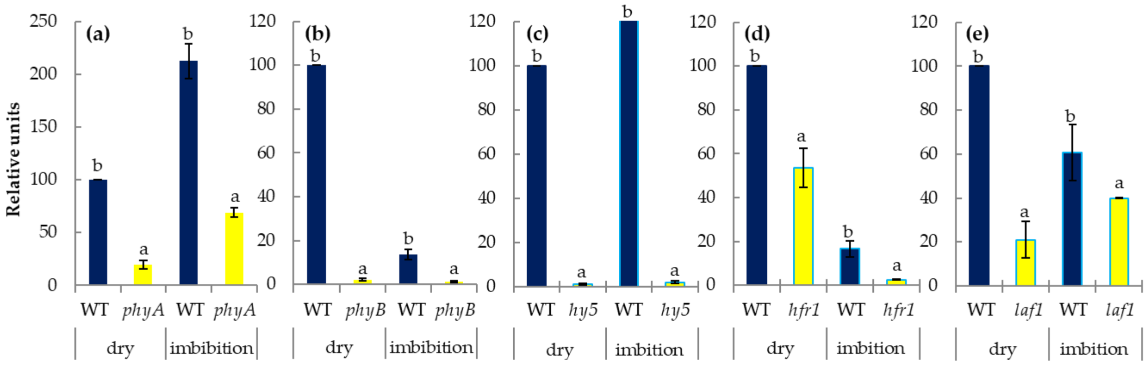

Supplementary Figure 3. The qRT-PCR analysis of relative expression of defined genes in dormant seeds (dry and imbibed for 6 h on light) of *Arabidopsis* T-DNA insertion mutants.

International Journal of Molecular Sciences

MDPI

Supplementary Table S2. Characteristic of primers used in qRT-PCR analysis of genes relative expression

Gene	Full gene name	Gene accession no.	NCBI number	L/R	Sequence 5′ --> 3′	Tm °C	Product size [bp]
**Reference genes**							
ACT7	*Actin 7*	**At5g09810**	NM_121018.3	**L**	GGAGCTGAGAGATTCCGTTG	58.1	197
				**R**	TCTTTGCTCATACGGTCAGC	57.9	
APC2	*Anaphase-Promoting Complex/Cyclosome 2*	**At2g04660**	NM_126495.2	**L**	GGAGGAGTTAAGCGCAATCA	57.9	154
				**R**	CACCAACTTGTCATGTCCCA	58.0	
HBT	*Hobbit*	**At2g20000**	NM_179663.2	**L**	ATCGATGAGAGCCCGTGAAA	59.2	181
				**R**	GGCTCTTGTGGCTGTGTTAC	58.1	
**Light signaling related genes**							
PHYA	*Phytochrome A*	**At1g09570**	NM_100828.3	**L**	CAAGGTCCGGATGATAGTTG	51.8	175
				**R**	AACTACAACCGCCATAACCA	49.7	
PHYB	*Phytochrome B*	**At2g18790**	NM_127435.3	**L**	AAAGAATCTGAGGCGGCTAT	49.7	199
				**R**	CTGCAATCTTAGCGTTCCAT	49.7	
CRY1	*Cryptochrome 1*	**At4g08920**	NM_116961.4	**L**	CAGCAGCGGAAGGAGAGAAA	60.0	232
				**R**	TTTCACCCGGAGTTACAGCCC	59.9	
CRY2	*Cryptochrome 2*	**At1g04400**	NM_100320.3	**L**	TTCGGGGAAATAAGCGTCAG	57.9	221
				**R**	TCAACATCAGCATCCCAAGG	57.9	
CRY3	*Cryptochrome 3*	**At5g24850**	NM_122394.3	**L**	GGAGAGATGCCAAGACAGGG	59.8	269
				**R**	TTGTGCTTGCTTGGGAATGC	59.9	
HY5	*Elongated Hypocotyl 5*	**At5g11260**	NM_121164.4	**L**	CGGAGAAAGAGAACAAGCGG	58.9	226
				**R**	CCACCACCTCCTCTCTTGTT	58.9	
HFR1	*Long Hypocotyl in Far-Red*	**At1g02340**	NM_100115.3	**L**	CTAAATCCGGCGAATCACAT	49.7	164
				**R**	GGAACCAAACCGTGAAGAGA	51.8	

HYH	*HY5-Homolog*	**At3g17609**	NM_180274.2	**L**	CACAAGAAGCACAAAACTGAGG	53.0	233
				**R**	TGCGCTGATACTCTGTTCCT	51.8	
LAF1	*Long After Far-Red Light 1*	**At4g25560**	NM_118688.3	**L**	CCCCTCCTTCGTCTTCATCA	58.8	181
				**R**	AGCCTTGGAATCTCAGGAGC	59.4	
CIB1	*Cryptochrome Interacting Basic Helix-Loop-Helix 1*	**At4g34530**	NM_119618.3	**L**	CACAGCATAGCAGAACGAGT	58.0	191
				**R**	GGCCTTGGATTCACAATTGC	57.9	
CIB5	*Cryptochrome Interacting Basic Helix-Loop-Helix 5*	**At1g26260**	NM_202184.2	**L**	TCGATGAAACTTTCGGCTGT	57.8	171
				**R**	TGAGGAAATGTTCGGTTGCA	58.0	
SUB1	*Calcium Ion Binding Protein SUB1*	**At4g08810**	NM_116951.1	**L**	GAAGGGCAAGCATCGAAGTA	57.9	230
				**R**	CTCCACACCTGAACCAATCC	58.1	
PP7	*Serine/Threonine Phosphatase 7*	**At5g63870**	NM_125782.3	**L**	CTGGTCTTGGTGGAATGGAC	58.1	171
				**R**	GAGGATCCGAGAAATCAGGC	57.8	
PHR2	*Photolyase/Blue Light Receptor 2*	**At2g47590**	NM_130327.3	**L**	ATCGCAGCGCTTGATCAATT	58.9	226
				**R**	CTGGTTTCCCTTGCTCAACC	59.0	
COP1	*Constitutive Photomorphogenic 1*	**At2g32950**	NM_128855.3	**L**	TCTCACAGTGAACAGCGAGT	58.9	199
				**R**	ATTCGCAGTCAACATCGTGG	58.9	
**Genes involved in ABA metabolism and signalling**							
ABA1	*ABA Deficient 1*	**At5g67030**	NM_126103.2	**L**	TGGCTGCAATTATGGCTTCC	51.8	217
				**R**	TCGGCTTTGTCAGTGAGTCT	51.8	
NCED6	*Nine-Cis-Epoxycarotenoid Dioxygenase 6*	**At3g24220**	NM_113327.2	**L**	AATGCGTGGGAAGAGAGAAC	57.9	205
				**R**	GGTTTATGTGACCCGCTTCT	57.9	
NCED9	*Nine-Cis-Epoxycarotenoid Dioxygenase 9*	**At1g78390**	NM_106486.2	**L**	CAATGATAGCCCACCCGAAA	57.9	239
				**R**	ACCTCTTATCATCTCCGGCA	57.9	
CYP707A2	*Cytochrome P450, Family 707, Subfamily A, Polypeptide 2*	**At2g29090**	NM_001202700	**L**	GCAGCAAGTGTACTGTCCTT	58.1	232
				**R**	GCTCACTTCCTGGACATGAG	57.9	
ABI3	*ABA Insensitive 3*	**At3G24650**	NM_113376.3	**L**	CATCTCCAGCTCCTGTCAAC	57.9	168

				R	CACCAGAAGAGTCGTCACAG	57.9	
ABI4	*ABA Insensitive 4*	**At2G40220**	NM_129580.1	L	GTTAGGGCAGGAACAAGGAG	57.8	155
				R	TATAACCCGGATCCAGACCC	57.9	
ABI5	*ABA Insensitive 5*	**At2G36270**	NM_129185.3	L	ATGATCAAGAACCGCGAGTC	58.1	221
				R	ATCAATGTCCGCAATCTCCC	58.0	
HAI1	*Highly ABA Induced PP2C Gene 1*	**At5g59220**	NM_125312.3	L	GTCACGAGCCATTGGAGATA	57.1	164
				R	AACACATTCGAACGACGCTA	57.9	
HAI2	*Highly ABA Induced PP2C Gene 2*	**At1g07430**	NM_100617.2	L	ATGTCACGAGCCATAGGAGA	57.9	236
				R	TCTTCTTCACTCCTCCTCCC	57.7	
HAI3	*Highly ABA Induced PP2C Gene 3*	**At2g29380**	NM_128492.2	L	CCGAGATTCCAAACGGTGAT	51.8	181
				R	CTCTTCTTCTACCGCACACC	53.8	
**Genes involved in GA metabolism and signaling**							
GA3ox1	*Gibberellin 3-Oxidase 1*	**At1g15550**	NM_101424.2	L	CTTGGGGTGCCTTCCAAATC	59.1	195
				R	AACCTTCGGACCACATTTGC	59.0	
GA3ox2	*Gibberellin 3-Oxidase 2*	**At1g80340**	NM_106683.1	L	CCAAGTTGATGTGGTTCGCA	59.0	176
				R	GAGGGTCGAGTCTGTATGGG	58.9	
GA2ox6	*Gibberellin 2-Oxidase 6*	**At1g02400**	NM_100121.4	L	AGCGAAGTGAGTGAGCTGAT	59.1	215
				R	GGGTCAGATGGGATTGGGAT	58.8	
GA2ox2	*Gibberellin 2-Oxidase 2*	**At1g30040**	NM_001036035.1	L	ATCCCCACATACAAACCGGT	59.0	193
				R	GCAAGCCGAAGAAGCCAATA	58.9	
GID1a	*GA Insensitive Dwarf1A*	**At3g05120**	NM_111384.3	L	TACTCTTTGTCGCAGGCTTG	51.8	239
				R	CCGATTCACCCGCTCTTAAA	51.8	
GID1b	*GA Insensitive Dwarf1B*	**At3g63010**	NM_116166.4	L	CCTCGAGCTAACCAAACCTC	53.8	151
				R	GACAACAACAACACCGCAAA	49.7	
GID1c	*GA Insensitive Dwarf1C*	**At5g27320**	NM_122614.3	L	TGTGCATAATGTCGCGGTAA	49.7	193
				R	ATGCTCTCTGTCTTCACCCT	51.8	

RGL1	*RGA-Like 1*	**At1g66350**	NM_105306.3	**L**	AAGCATGCTCTCGGATCTTG	51.8	163
				**R**	AGAGCGCGTAGAGGATAACT	51.8	
RGL2	*RGA-Like 2*	**At3g03450**	NM_111216.2	**L**	ACCGGAGTTAGACTTGTCCA	51.8	176
				**R**	CGGTAAATACGACGAGCCAA	51.8	
RGL3	*RGA-Like 3*	**At5g17490**	NM_121755.2	**L**	ACTTACACTCTCCAGGTCCC	53.8	239
				**R**	GTTATCGTCTCCACCACCAC	53.8	
XERICO	*RING/U-box superfamily protein*	**At2g04240**	NM_201687.1	**L**	GCAAGAACAGGCAGACAA	48.9	172
				**R**	GGCACAACAACAAGAGGAG	51.1	
KAO2	*Arabidopsis thaliana Ent-kaurenoic acid axidase 2*	**At2g32440**	NM_001202728	**L**	CAGGGAAGCAAAGAGTGATG	58.4	181
				**R**	CAAACCAAAAGGAAGGAATG	57.1	

International Journal of
Molecular Sciences

MDPI

© 2019 by the authors. Submitted for possible open access publication under the terms and conditions of the Creative Commons Attribution (CC BY) license (http://creativecommons.org/licenses/by/4.0/).
